# Integrating microbial source tracking with quantitative microbial risk assessment to evaluate site specific risk based thresholds at two South Florida beaches

**DOI:** 10.3389/fmicb.2023.1210192

**Published:** 2023-10-12

**Authors:** Anna Gitter, Maribeth Gidley, Kristina D. Mena, Alesia Ferguson, Christopher Sinigalliano, Anthony Bonacolta, Helena Solo-Gabriele

**Affiliations:** ^1^Department of Epidemiology, Human Genetics and Environmental Sciences, University of Texas Health Science Center Houston School of Public Health, El Paso, TX, United States; ^2^Cooperative Institute for Marine and Atmospheric Studies, University of Miami, Miami, FL, United States; ^3^Atlantic Oceanographic and Meteorological Laboratory, National Oceanic and Atmospheric Administration, Miami, FL, United States; ^4^Department of Built Environment, North Carolina Agricultural and Technical State University, Greensboro, NC, United States; ^5^Department of Marine Biology and Ecology, University of Miami, Miami, FL, United States; ^6^Institut de Biologia Evolutiva (CSIC-Universitat Pompeu Fabra), Barcelona, Catalonia, Spain; ^7^Department of Chemical, Environmental, and Materials Engineering, University of Miami, Coral Gables, FL, United States

**Keywords:** fecal indicator bacteria, microbial source tracking, quantitative microbial risk assessment, beach, child, exposure

## Abstract

Quantitative microbial risk assessment (QMRA) can be used to evaluate health risks associated with recreational beach use. This study developed a site-specific risk assessment using a novel approach that combined quantitative PCR-based measurement of microbial source tracking (MST) genetic markers (human, dog, and gull fecal bacteria) with a QMRA analysis of potential pathogen risk. Water samples (*n* = 24) from two recreational beaches were collected and analyzed for MST markers as part of a broader Beach Exposure And Child Health Study that examined child behavior interactions with the beach environment. We report here the measurements of fecal bacteria MST markers in the environmental DNA extracts of those samples and a QMRA analysis of potential health risks utilizing the results from the MST measurements in the water samples. Human-specific *Bacteroides* was enumerated by the HF183 Taqman qPCR assay, gull-specific *Catellicoccus* was enumerated by the Gull2 qPCR assay, and dog-specific *Bacteroides* was enumerated by the DogBact qPCR assay. Derived reference pathogen doses, calculated from the MST marker concentrations detected in recreational waters, were used to estimate the risk of gastrointestinal illness for both children and adults. Dose–response equations were used to estimate the probability of the risk of infection (P_inf_) per a swimming exposure event. Based on the QMRA simulations presented in this study, the GI risk from swimming or playing in water containing a mixture of human and non-human fecal sources appear to be primarily driven by the human fecal source. However, the estimated median GI health risk for both beaches never exceeded the U.S. EPA risk threshold of 32 illnesses per 1,000 recreation events. Our research suggests that utilizing QMRA together with MST can further extend our understanding of potential recreational bather risk by identifying the source contributing the greatest risk in a particular location, therefore informing beach management responses and decision-making.

## 1. Introduction

Time spent at the beach playing in the sand or swimming along the shoreline is a common recreational activity in Florida and many other coastal regions. However, activities at the beach may expose individuals to various contaminants found in that environment. The presence of potentially infectious bacteria in the beach environment can pose a health risk for beach goers through either direct skin contact, inhalation, or ingestion. As part of the BEACHES project (Beach Exposure And Child Health Study) during the summer of 2018, children were observed and documented while at play on beaches in Miami, Florida. The BEACHES study documented for the first-time specific exposure factors for children engaged in beach play activities (Ferguson et al., 2020, 2021a,b; Tomenchok et al., 2020) relative to beach characteristics. Water ingestion was identified as a possible exposure pathway following observations of children wading in seawater.

Historically, recreational water quality management has been targeted for monitoring concentrations of culturable fecal indicator bacteria (FIB), specifically *Escherichia coli* in freshwater and enterococci in marine water, to protect bather health. Beach advisories are issued when levels of bacteria in waters exceed a threshold level of FIB. At marine sites in the U.S., advisories are based on the levels of enterococci, since they are typically found in human feces and sewage (Fleisher et al., 2010). Studies have linked elevated enterococci levels at beaches and increased human health risks to recreational bathing activities for beaches impacted by point sources of sewage discharge (U.S. EPA, 2012b). States promulgate regulatory bacterial water quality criteria, as well as recommend “Beach Action Values” (BAV) to guide beach manager decisions for issuing warning advisories. These State promulgated regulatory criteria are based upon the national recreational water quality criteria recommended by the U.S. EPA. In the state of Florida, beach monitoring is coordinated through the Florida Department of Health (FDOH) Florida Healthy Beaches Program (FHBP). The bacterial water quality regulatory criteria of the state are implemented via the Florida Administrative Code (FAC), based on the U.S. EPA’s national water quality recommendations (U.S. EPA, 2012b). The threshold for the monthly viable enterococci geometric mean in recreational waters is 35 colony forming units (CFU) per 100 mL (FL DEP, 2022, 62–302.530, FAC). The U.S. EPA has historically conducted a series of epidemiological studies at recreational beaches and has determined that this level of viable enterococci exposure in marine bathing water (if originating from a known point source of human sewage contamination) is associated with approximately 32 illnesses per 1,000 bathers (U.S. EPA, 2012b). Furthermore, there is also the recommendation for a single-sample Statistical Threshold Value (STV) in conjunction with a BAV. The STV is based on the results from single grab samples during water quality monitoring rather than a monthly geometric mean of multiple repeated samples, and beach warning advisories are recommended when the BAV of 70 CFU per 100 mL of viable enterococci is exceeded.

It should be recognized, however, that all of these criteria and action values are based upon the culture-based enumeration of live enterococci. There is also the presumption that all the detected enterococci are from human sewage sources, so as to be conservatively protective of public health. However, enterococci in the beach environment can come from a wide variety of sources including both treated and untreated sewage, human bather shedding (Elmir et al., 2007, 2009; Arnold et al., 2017), non-human animals (Wright et al., 2009; Sinigalliano et al., 2010; Boehm et al., 2013), and may even persist or regrow in the beach environment such as in beach sediments or seaweed wrack (Abdelzaher et al., 2010; Badgley et al., 2010; Whitman et al., 2014; Solo-Gabriele et al., 2016; Abdool-Ghany et al., 2022). These various sources of FIB may result in different public health risks, however the culture-based methods used for regulatory beach monitoring do not have a source tracking capability to distinguish FIB sources. While epidemiological studies have linked swimmer gastrointestinal illnesses with an increase in FIB concentrations, there are limitations to solely relying on these indicators to evaluate recreational water quality (Wade et al., 2003; Zmirou et al., 2003; McKee and Cruz, 2021).

Microbial source tracking (MST) has been identified as a method to supplement water quality management, given it can assist in identifying specific sources of fecal contamination. MST is a DNA-based technology that enables the water management community to determine whether humans or other animal species are responsible for microbial fecal contamination in an environmental sample. A variety of methods for molecular MST of FIB have been developed, tested, and deployed, and applications for MST in water quality management are becoming increasingly common (Harwood et al., 2014). Many species of animals have host-specific strains of fecal bacteria with unique diagnostic DNA sequences, which can be targeted for detection and enumeration in environmental DNA extracts. While different MST technologies are available, the gene amplification assays based upon quantitative PCR (qPCR) or on digital PCR (dPCR or ddPCR) are especially popular, and many such qPCR MST assays have been developed and validated. This type of approach can be highly effective when integrated into a multi-tool, multi-tiered strategy for water quality assessment, as described in the California Microbial Source Identification Manual (Griffith et al., 2013).

Risk based thresholds (RBTs) (molecular marker concentrations which correspond to a health risk probability of 0.032 for a gastrointestinal illness) have been proposed to assist beach managers in identifying unsafe conditions of water quality. Quantitative microbial risk assessment (QMRA) is a tool that can be used to evaluate human health risks from exposure to microbial contaminants to inform RBT development and applicability. The framework consists of four phases – hazard identification, exposure assessment, dose–response, and risk characterization – and collectively, can estimate the human health risks associated with exposure to specific microorganisms. There have been previous QMRA studies that suggested RBTs for molecular markers, specifically targeted for human and gull fecal sources. The most recent RBT proposed for the human-associated *Bacteroides* molecular marker HF183, assuming the sewage contamination is of unknown age, is 525 copies/100 mL; however, the proposed RBT for the gull molecular marker of unknown fecal age is much higher at 200,000 copies/100 mL. If both molecular markers are present (if human and gull fecal waste are co-occurring), the HF183 RBT marker value should be adjusted ranging from 1 to 525 copies/100 mL (Boehm and Soller, 2020) in order to correspond to the accepted RBT.

In the current study, environmental MST markers were quantified in recreational marine waters, and a subset of those markers were then utilized to develop a site-specific risk assessment. To the authors knowledge, this is the first time that a QMRA is developed for a recreational beach that utilizes MST marker levels for dog markers, in addition to human and gull markers. The intention of the work is to provide a framework to assess recreational beach risk based upon multiple sources of FIB measured in marine water used for swimming. The results of such QMRAs can be used as a starting point for beach managers to evaluate recreational water quality and inform beach management decision-making when beaches are impacted by humans, dogs, and gulls.

## 2. Materials and methods

### 2.1. Study area and study design

**Figure 1:**
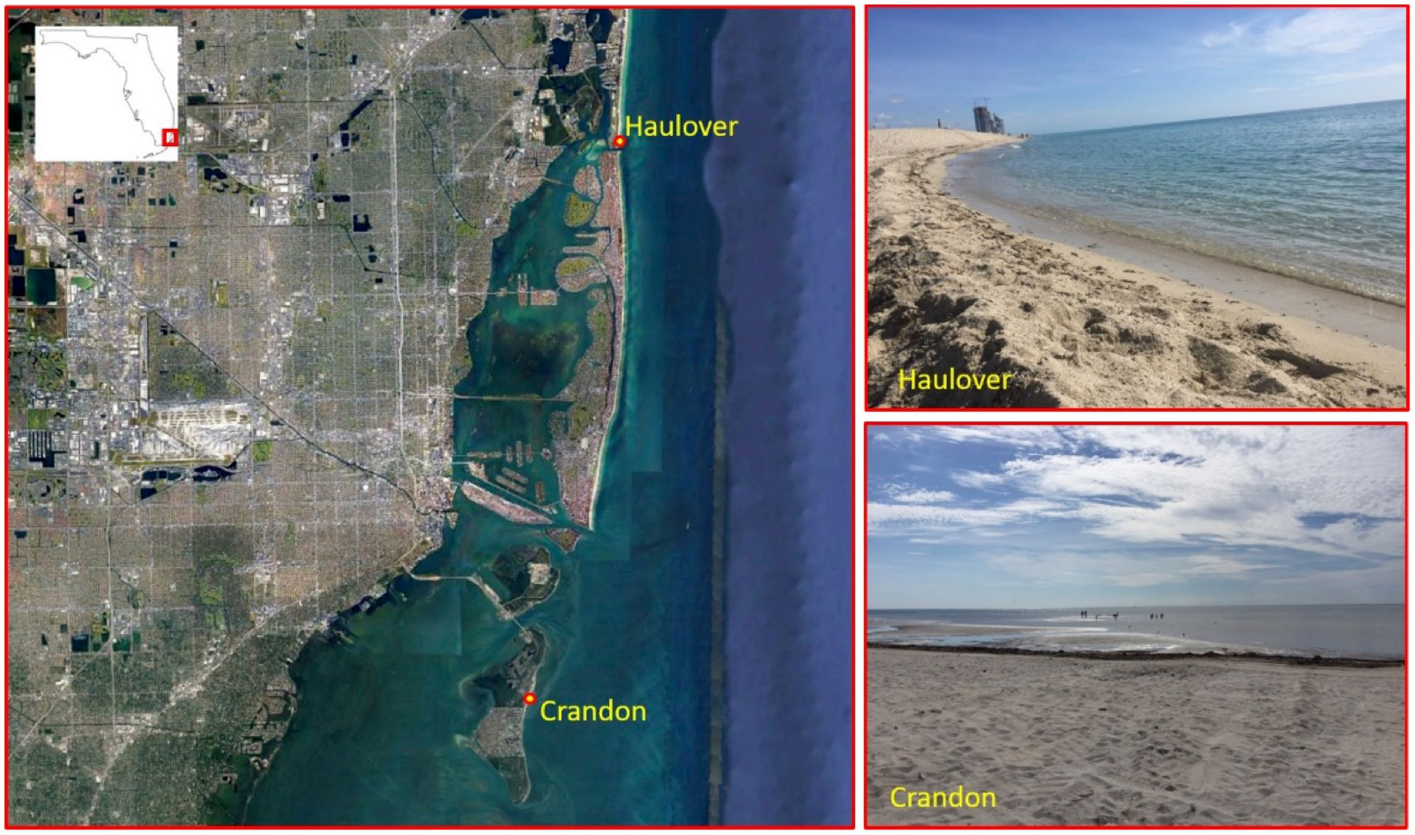
Location and photographs of study beaches in Miami Dade County, Florida, for the beach exposure and child health study.

The integrated QMRA-MST study described in this paper is part of a much larger BEACHES project, which aimed to collect activity patterns for children on the beach, quantify oil spill concentrations in the nearshore environment [as related to the 2010 Deepwater Horizon Oil Spill (Montas et al., 2020; Xia et al., 2020, 2021; Montas et al., 2022a,b)], and estimate chemical and bacterial exposure and health risks for young children. The overall study design, analysis of child-beach interaction behavior, and the predicted chemical exposure risk assessments are reported elsewhere (Altomare et al., 2021; Ferguson et al., 2021a). We report here the environmental MST data and the microbial risk assessment component of this BEACHES project. Two sub-tropical South Florida marine recreational beaches in the Miami region – Crandon Park Beach (CPB) and Haulover Beach (HB) – were utilized in this study (Figure 1). Both beaches are considered non-point source. CPB often has dense human usage on weekends, but medium usage during weekdays, while birds may frequently be observed on the beach depending on the time of year. HB also has dense human populations on weekends, but is sparser during weekdays, as well as a sparse bird population. Neither beach officially allows dogs in the regions sampled, however there is a designated dog beach near the HB location.

Water samples (*n* = 24) were aseptically collected at CPB and HB during the BEACHES study. Both CPB and HB were sampled on 4 consecutive days at 3 time points each day (morning before the start of the child video observations and data collection, mid-day during the child observations, and late afternoon following the conclusion of the child observations) during the period of June 21st, 2018 to June 24th, 2018 (Thursday to Sunday) and June 27th, 2018 to June 30th, 2018 (Wednesday to Saturday), respectively. Both beaches are part of the Florida Healthy Beaches Program and are routinely monitored once a week by Miami-Dade County for levels of culturable enterococci in the swim zone. However, during the sampling days of this study no samples collected as part of the Florida Healthy Beaches Program for either beach exceeded the bacteriological water quality guideline level. Thus no advisories were posted at the time of sampling for either of these beaches.

### 2.2. Sample collection and preservation

Upon collection, all water samples were placed in a cooler with ice and were transported back to the laboratory within 6 h for immediate pre-processing and preservation of DNA. The water samples (1 liter in volume) were collected up-current in sterile polypropylene bottles from approximately 1 cm below the surface at knee depth. At the lab, water samples were filtered onto 0.45 μm pore-sized mixed cellulose ester 47 mm diameter filters (GN-6 Metricel, Pall Corp), filtering 1 liter or until filter clogging (and recording actual filtered volume). Filter samples were aseptically rolled and transferred into sterile 2 mL polypropylene tubes. Filters were then stored at −80°C until extraction.

### 2.3. Extraction and purification of environmental DNA

For each sample, the corresponding frozen filter was aseptically transferred to a “Lysing Matrix E” bead-beat homogenization tube from the FastDNA Spin Kit for Soil (MPBiomedicals) along with 1 mL of lysis buffer from the kit as per manufacturer’s instructions. All water filters were homogenized by 2 rounds each of bead-beating in a FastPrep-24 homogenization instrument (MPBiomedicals) with an impact speed setting of 6.0 m/s for 60 s each (with a 5-min cool-down period between each round of bead-beating). Before homogenization, the lysis buffer was amended with Chum Salmon DNA at 0.2 μg/mL as a Sample Processing Control (SPC, as per EPA Method 1,696). The lysate tubes were centrifuged at >14,000 x g for 15 min to pellet debris, then the lysate was transferred and purified using the FastDNA Spin Kit for Soil as per manufacturer instructions and eluted in a final volume of 100 μL with the kit’s elution buffer. The eluted purified eDNA samples were divided into replicate aliquots and stored frozen at −20°C until qPCR analysis.

### 2.4. MST qPCR analysis

The relative abundances of FIB in the water samples were enumerated by molecular MST using qPCR assays specific for: [1] the general enterococci 23S rRNA gene marker “Entero1A” assay by EPA method 1611.1 (U.S. EPA, 2012a); [2] the human host specific *Bacteroides* 16S rRNA gene marker “HF183 – TaqMan” assay by EPA method 1696 (U.S. EPA, 2019a); [3] the human host specific *Bacteroides* gene marker “HumM2” assay by EPA method 1697 targeting *Bacteroides*-like cell surface protein genes (U.S. EPA, 2019b); [4] the dog host specific *Bacteroides* 16S rRNA “DogBact” marker assay as per the California Microbial Source Identification Manual (Griffith et al., 2013) and; [5] the Gull associated 16S rRNA *Catellicoccus marimammalium* gene marker “Gull2” assay as per the California Microbial Source Identification Manual (Griffith et al., 2013). This study quantified the environmental concentration of *Catellicoccus marimammalium* using the Gull2 marker, while available dose data needed for the QMRA for gull feces impacts quantified *Catellicoccus marimammalium* using the LeeGull MST marker. We presumed in this study that the Gull2 and LeeGull MST markers provide consistent results when utilized in the QMRA because both the Gull2 and LeeGull MST markers target the same region of *Catellicoccus marimammalium*. Descriptions regarding the MST qPCR analysis, including the primers, probes, and positive controls (Supplementary Table S1), and modifications that were made are summarized in the Supplementary materials. The quality control and assurance metrics for the qPCR standard curves are further described and summarized in Supplementary Table S2.

### 2.5. QMRA

A subset of the markers – HF183, Gull2, and DogBact – were further utilized in a QMRA to estimate the human health risks associated with reference pathogens representative of each marker and to compare these risks to previously published RBTs (Boehm and Soller, 2020). The human associated *Bacteroides* “HF183” and gull associated *Catellicoccus marimammalium* “Gull2” markers were selected since they have consistently been identified and utilized to detect source-specific fecal pollution and inform QMRA studies (Boehm et al., 2013; Brown et al., 2017b; Boehm and Soller, 2020). The dog associated *Bacteroides* “DogBact” was also included due to the frequency by which dog fecal pollution has been detected at beaches and increased interest to provide educational interventions regarding dog presence and dog waste management at beaches (Oates et al., 2017).

#### 2.5.1. Hazard identification and exposure assessment

Reference pathogens for each of the three MST gene markers were selected based upon their environmental prevalence and health risks in recreational waters and are often applied in QMRA studies (Soller et al., 2010, 2017; Whiley et al., 2013; Brown et al., 2017b; Boehm and Soller, 2020; Owens et al., 2020). The specific reference pathogens used to represent the different fecal sources – human, gull, and dog feces – were identified from previous studies (Chaban et al., 2010; Brown et al., 2017b; Boehm and Soller, 2020). Bather shedding from enterococci on skin is believed to be the primary contributor of microbial pollution at these particular beaches (Li et al., 2021) due to the lack of permitted wastewater treatment facilities discharging in the area. Given the lack of information regarding specific pathogens associated with bather shedding, microbial pathogens found in human sewage were used in the QMRA analysis. The reference pathogens representing human sewage – norovirus, adenovirus, *Cryptosporidium*, *Giardia*, *Campylobacter*, *Salmonella* spp. and *E. coli* O157:H7 – have been used in several QMRAs assessing the health risks associated with recreational waters (U.S. EPA, 2010, 2014; Soller et al., 2010). Gull feces have been represented by *Salmonella* and *Campylobacter* in other QMRA studies which integrated non-human fecal sources (Schoen and Ashbolt, 2010; Soller et al., 2014; Brown et al., 2017b). Dog fecal waste has not been assessed in a QMRA study before, but *Campylobacter* is identified as a pathogen of concern for pet owners and can result in pet-associated human campylobacteriosis (Parsons et al., 2010; Gras et al., 2013; Whiley et al., 2013; Acke, 2018). All reference pathogens selected have the health endpoint of a gastrointestinal infection and illness, commonly known as gastroenteritis. A reference pathogen dose can be calculated from the MST marker concentration detected in recreational waters for the three different fecal sources (Equation 1; Soller et al., 2010; Brown et al., 2017b; Gitter et al., 2020).


(1) $$dose_{RP}^{s}=\frac{C_{MST}}{F_{MST}^{S}\times 100}\times R_{RP}^{s}\times P_{s}\times V$$

where *S* represents each fecal source as indicated by the MST markers (human, gull, and dog); MST indicates each MST marker (HF183, Gull2, and DogBact); *RP* refers to reference pathogen; *C_MST_* is the concentration of the specific MST marker as measured in the environment (copies/100mL); $F_{MST}^{S}$ is the concentration of the specific MST marker in sewage or feces for each fecal source (copies/mL or copies/g); $R_{RP}^{S}$ is the concentration of the reference pathogen in the sewage or feces of each fecal source (n/g or n/L); *P*_S_ is the fraction of human-infectious species or serotypes; and *V* is the volume of water ingested per each swimming event (mL). A conversion factor of 0.001 is needed when calculating the reference pathogen dose for the sewage source since the $R_{RP}^{S}$ is measured in L and the *V* is measured in mL.

The exposure event of interest in this QMRA is of seawater ingestion while recreating at either of these two beaches. Incidental ingestion of ambient seawater for a singular swimming event (including wading, swimming, and/or playing) was distinguished between both adults and children. Ingestion values were retrieved from a previous study evaluating environmental exposures to water at beaches across 12 locations (with 68,000 participants) in the U.S. Documented ingestion values for both adults and children followed a normal distribution, with mean values of 32.3 mL and 67.7 mL, respectively (DeFlorio-Barker et al., 2018). Mean values for time spent in water, which is included in the incidental ingestion volume, was 121.4 min for children and 66.9 min for adults (DeFlorio-Barker et al., 2018).

Concentrations of HF183, Gull2, and DogBact in raw sewage and feces were obtained from the literature (Shanks et al., 2010; Ervin et al., 2014; Brown et al., 2017a). Since pathogens from nonhuman fecal sources are known to not be as infectious to outside hosts, fractions for pathogenicity for humans were used for the gull and dog pathogens. In brief these fractions include 0.01–0.4 for gulls, 0.01–0.1 for dogs, and 1 (100%) for human-source pathogens (García-Aljaro et al., 2005; Gras et al., 2013). The input parameters utilized in the dose equation – environmental concentrations of MST markers, concentrations of MST markers and pathogens in raw sewage/feces, volume of water ingested, and fraction of pathogenic species – are listed in Supplementary Table S3 (Hurst et al., 1988; Lévesque et al., 2000; Koivunen et al., 2003; Lemarchand and Lebaron, 2003; Crockett, 2007; U.S. EPA, 2009; Hewitt et al., 2011; Kitajima et al., 2014; Nasser, 2015; Yang et al., 2015; Eftim et al., 2017; Schoen et al., 2017). This QMRA evaluates the individual risk of exposure in a static model and does not consider immunity or secondary transmission.

#### 2.5.2. Dose response

Dose–response equations were utilized to estimate the probability of the risk of infection (*P_inf_*) per swimming event (an assumed amount of time spent recreating in water while at the beach) (Table 1) and had been previously developed using feeding studies and outbreak data. *Salmonella*, *Campylobacter* and *E. coli* O157:H7 have been fit to a Beta-Poisson dose–response model (Medema et al., 1996; Haas et al., 1999; Teunis et al., 1999, 2008). An exponential model has been fit to data to estimate the dose–response relationships for *Cryptosporidium*, *Giardia,* and adenovirus (Couch et al., 1969; Rose and Gerba, 1991; Eisenberg et al., 1996; Crabtree et al., 1997; U.S. EPA, 2006), while a Fractional Poisson model has been used for norovirus (Messner et al., 2014; Van Abel et al., 2017b).

**Table 1:** Dose–response relationships and morbidities for each reference pathogen.

Pathogen	Probability of infection	Morbidity ratio	References
*Salmonella* spp.	1-(1 + dose/2884)^−0.3126^	0.17–0.4^a^	Haas et al. (1999), Teunis et al. (1999)
*Campylobacter*	1-(1 + (dose/7.59))^−0.145^	0.1–0.6^a^	Medema et al. (1996)
*E. coli* O157:H7	1-(1 + (dose/48.8))^−0.248^	0.2–0.6^a^	Teunis et al. (2008)
*Cryptosporidium*	1-exp (−0.09*dose)	0.3–0.7^a^	U.S. EPA (2006)
*Giardia*	1-exp (−0.01982*dose)	0.2–0.7^a^	Rose and Gerba (1991), Eisenberg et al. (1996)
Norovirus	0.72*(1-exp (−dose/1))^c^	0.3–0.8^a^	Messner et al. (2014), Van Abel et al. (2017b)
Adenovirus	1-exp (−dose *0.4172)	0.5^b^	Couch et al. (1969), Crabtree et al. (1997)

^a^Uniform distribution (minimum, maximum); ^b^Point estimate; ^c^Full particle disaggregation is assumed with μ = 1.

For the norovirus dose–response model, a conservative version of the model was utilized assuming full particle disaggregation (Regli et al., 1991; Haas et al., 1999; Vergara et al., 2016). There is a lack of consensus regarding which norovirus dose–response model is most appropriate for specific environmental situations. However, for recreational waters, which tend to have higher norovirus concentrations than untreated drinking water, most norovirus dose response models predict similar values for the probability of infection. The other dose-relationships presented in Table 1 have all been used in previous recreational or drinking water QMRA studies.

The probability of illness (*P_ill_*) for each pathogen was estimated by multiplying the *P_inf_* and the morbidity of each respective pathogen. When applicable, the morbidity or proportion of infections that result in illness was described as a value drawn from a uniform distribution. The probability of illness due to exposure to a combination of the three different fecal sources, as represented by reference pathogens, was estimated using Equation 2. For mixed sources that shared the same reference pathogens, the $dos{e_{RP}^{S}}^{”}$ was calculated independently for each fecal source and then summed together to find the total *dose_RP_*. The cumulative risk of illness combines statistically independent exposures (Regli et al., 1991; Soller et al., 2010).


(2) $$P_{\mathrm{ill}}^{s}=1-\underset{RP}{\prod }\left(1-P_{\mathrm{ill},RP}\right)$$

Crystal Ball Pro® Software (Oracle Corp., Austin, TX) was used to conduct the Monte Carlo simulations (10,000 simulations for each exposure scenario). For each simulation, the QMRA model used input parameters that are described by statistical distributions (when appropriate) to include inherent variability in the model (Supplementary Table S3). Probability plots were developed for the interval censored MST marker concentrations using Minitab® software (Minitab LLC, State College, PA, USA) (RRID:SCR_014483). Utilizing maximum likelihood estimation (MLE), the datasets were fit to the Weibull, lognormal, exponential, loglogistic, and normal distributions. Best fits for the datasets and the fitted distributions were based upon the Anderson-Darling (A-D) and Kolmogorov–Smirnov (K-S) tests. Probability plots and MLE for best fitted distributions based upon the A-D and K-S tests were also conducted using a substitution technique for the non-detects. Non-detect values were substituted with ½ the detection limit (25 copies/100 mL). Details about the derivation of the detection limit are provided in the Supplementary material. Graphs were developed using GraphPad Prism® (GraphPad, San Diego, CA) (RRID:SCR_002798). For abbreviations, interval censored datasets fitted to a probability distribution were identified as INT and datasets that had non-detects substituted with ½ the detection limit and fitted to a distribution are identified as DL.

## 3. Results

### 3.1. qPCR of microbial source tracking

**Figure 2:**
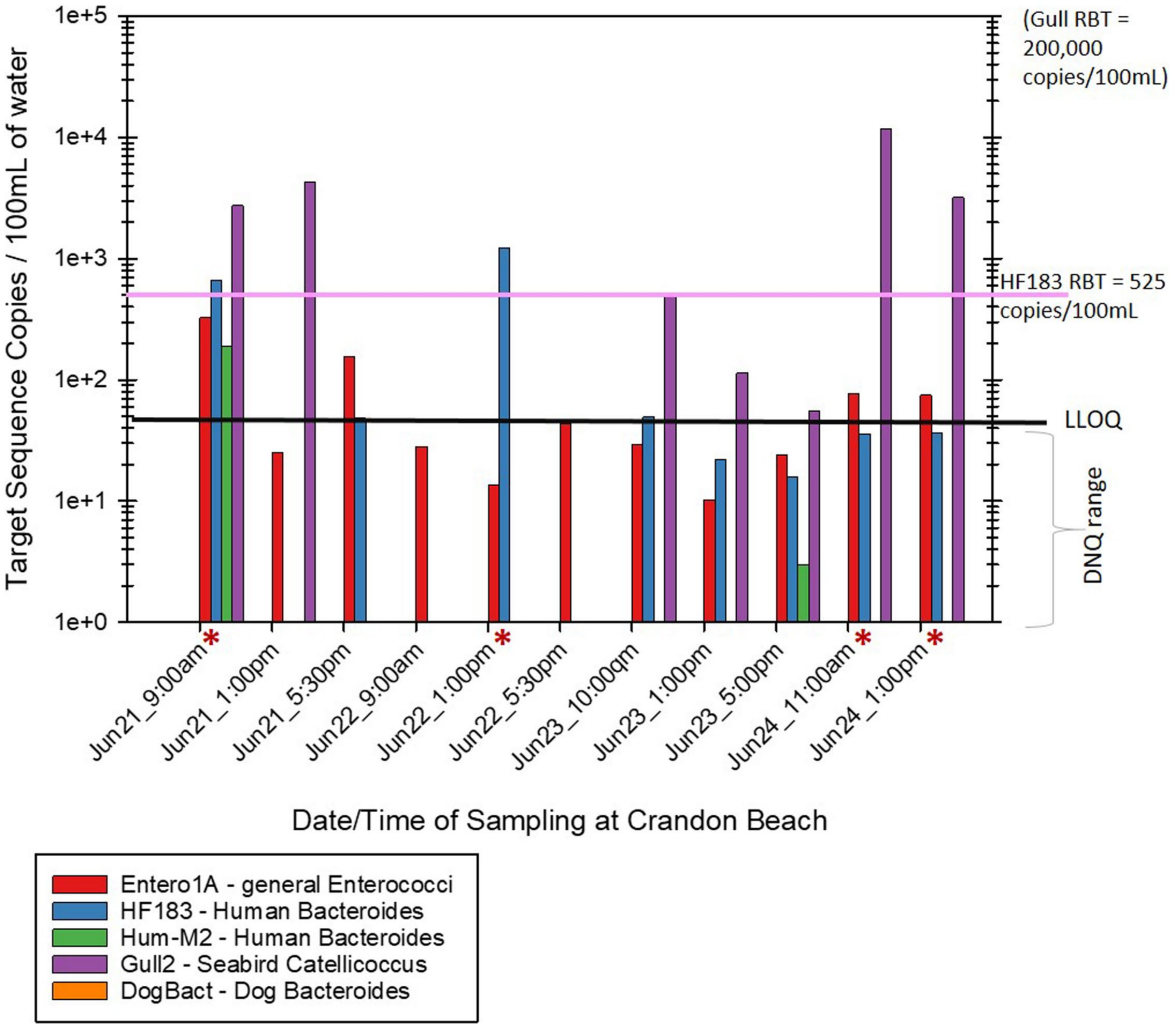
Abundance by date and time of MST host-source-specific fecal bacterial gene markers in bathing water at Crandon Park Beach. Red asterisks by the sample date/time indicate samples that exceeded the calculated 32/1000 illness rate based on the combined levels of HF183 + Gull markers. The black line labeled “LLOQ” indicates the environmental Lower Limit of Quantitation of 50 target copies/100 mL for the listed MST gene markers.

**Figure 3:**
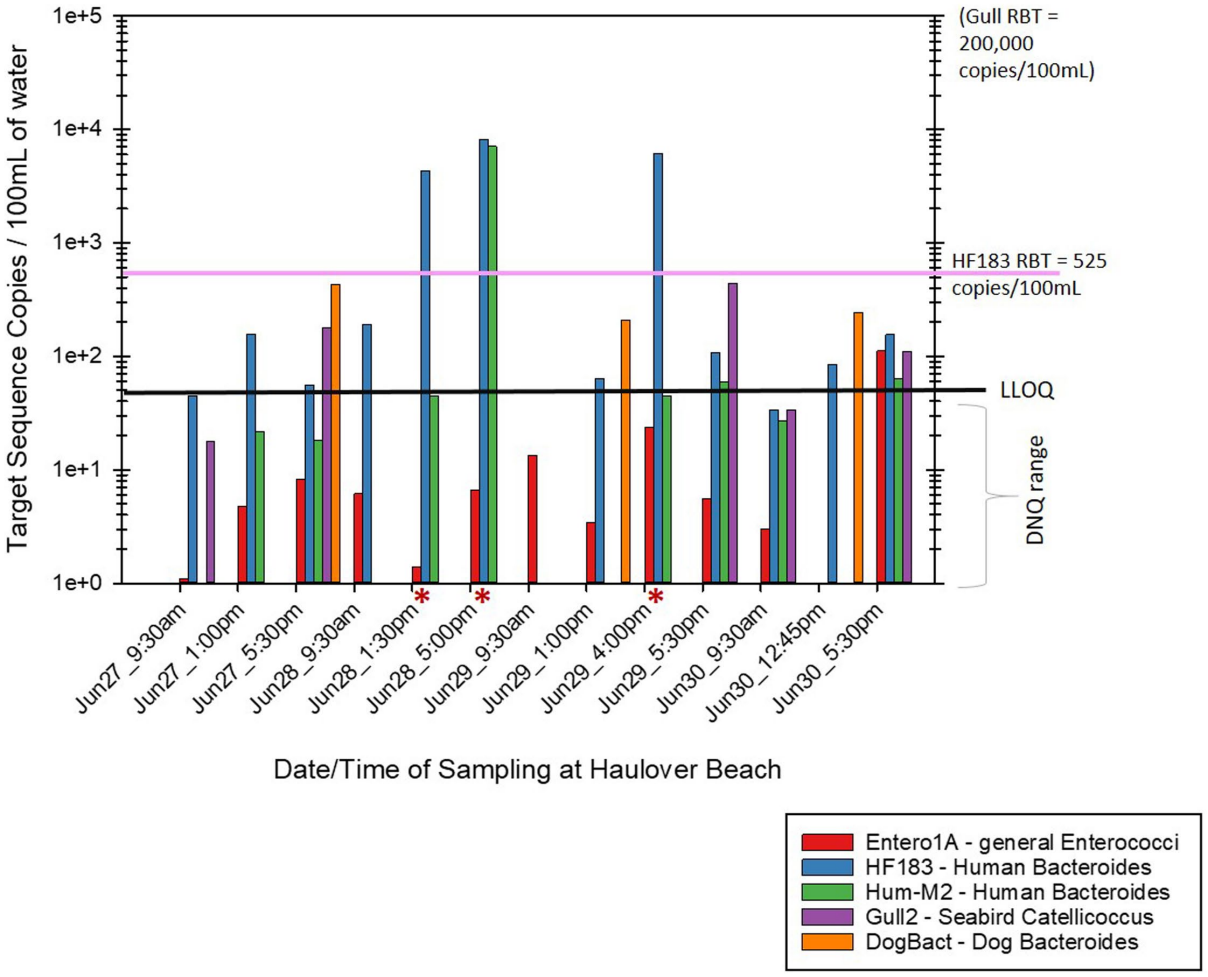
Abundance by date and time of MST host-source-specific fecal bacterial gene markers in bathing water at Haulover Beach. Red asterisks by the sample date/time indicate samples that exceeded the calculated 32/1000 illness rate based on the combined levels of HF183 + Gull markers. The black line labeled “LLOQ” indicates the environmental Lower Limit of Quantitation of 50 target copies/100 mL for the listed MST gene markers.

**Figure 4:**
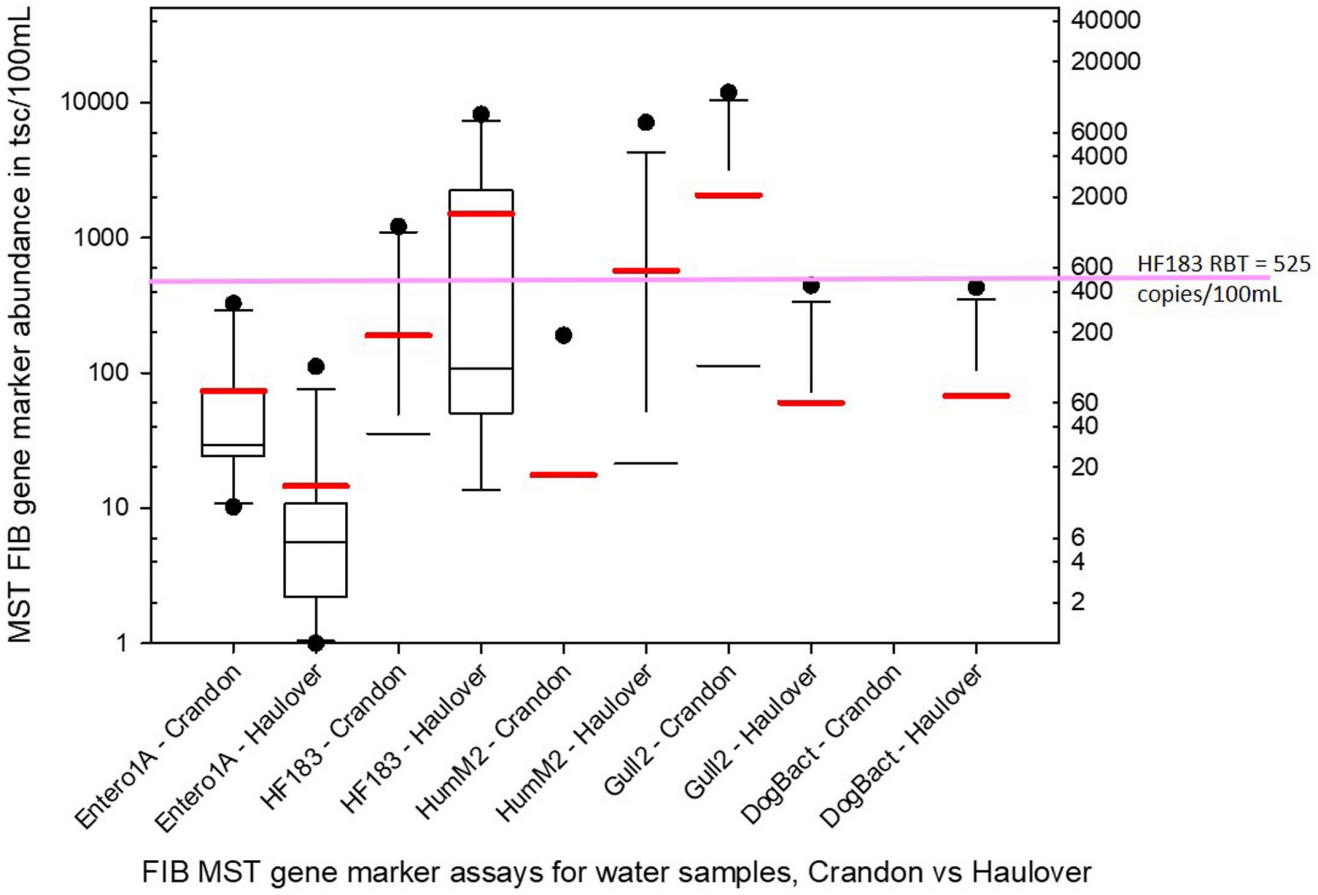
Boxplots of the statistical distribution of the cumulative abundance for all sample dates/times of each of the 5 MST fecal bacteria markers as measured by qPCR for Crandon Beach and Haulover Beach. The red bar associated with each plot marks the mean whereas the black line within the box marks the median. Black circles represent outliers. Note that for some combinations of marker/beach the range between the 25th and 75th percentiles are too narrow to generate a visible box at this scale and is just indicated by the median bar.

Of the human associated MST gene markers quantified within the water samples, the levels of the HF183 human *Bacteroides* marker were often much higher than the levels of Hum-M2 human *Bacteroides* marker (Figures 2, 3). The levels of human-source fecal bacteria marker were substantially higher and more frequent at HB than at CPB, whereas the levels of non-host-specific general Entero1A marker were frequently much higher at CPB than at HB (Figures 2–4). While there were many low-level detects of the HF183 human-associated fecal *Bacteroides* marker, overall, there was relatively little exceedance at either beach of the 525 copies/100 mL RBT for recreational waters contaminated with sewage of uncertain age (as per Boehm and Soller, 2020). Only 3 samples from HB and 2 samples from CPB exceeded this recommended exposure threshold for human fecal contamination of uncertain age. The dog-associated fecal *Bacteroides* marker was rarely seen at either beach during the sampling days of this study, and when seen it was in relatively low abundance. The detection of bird fecal marker contamination was highly variable at both beaches but contributions of higher levels of gull associated *Catellicoccus* marker were seen on many days at both beaches, with no apparent pattern. No samples exceeded the Gull-only RBT of 200,000 copies/100mL. However, 2 samples from CPB (both on June 24th) showed a combined level of >30 copies/100mL of the HF183 marker + >3,000 copies/100mL of Gull marker. These markers together exceed the proposed RBTs suggested by Boehm and Soller (2020), even though neither exceeded the HF183-only RBT or the Gull-only RBT. It is important to note that the QMRA analysis conducted in this study includes all MST marker concentrations that were quantified during sampling. It is likely that birds, which are frequently present, especially on CPB, were the primary contributors of animal-associated fecal contamination of these beaches during the study.

Contributions from dog sources, as neither site officially allows dogs, was relatively minimal, though the minor presence of dog contamination at HB may be due to the presence of a nearby dog beach. Additional qPCR MST analyses of sand and seaweed wrack samples collected from both beaches are described in Supplementary Figures S1–S6 (although the QMRA analysis only focuses on the water samples).

### 3.2. Health risk estimates using QMRA

#### 3.2.1. Risks associated with each fecal source

The risk of illness corresponding to each fecal source was evaluated utilizing the concentrations of the MST markers in water samples and assuming an exposure scenario that included recreation (swimming, wading, playing, etc.) in marine water for both children and adults. The risks associated with each fecal source, as detected at both HB and CPB, were computed and compared with the U.S. EPA risk threshold of 0.032 (U.S. EPA, 2012b). It is important to note that since CPB did not have any detected dog fecal contamination (as indicated by non-detects for the DogBact MST marker), two scenarios were evaluated for this beach: (a) no DogBact MST marker present and (b) DogBact MST marker concentrations of ½ the detection limit, assuming dog fecal contamination is present.

**Figure 5:**
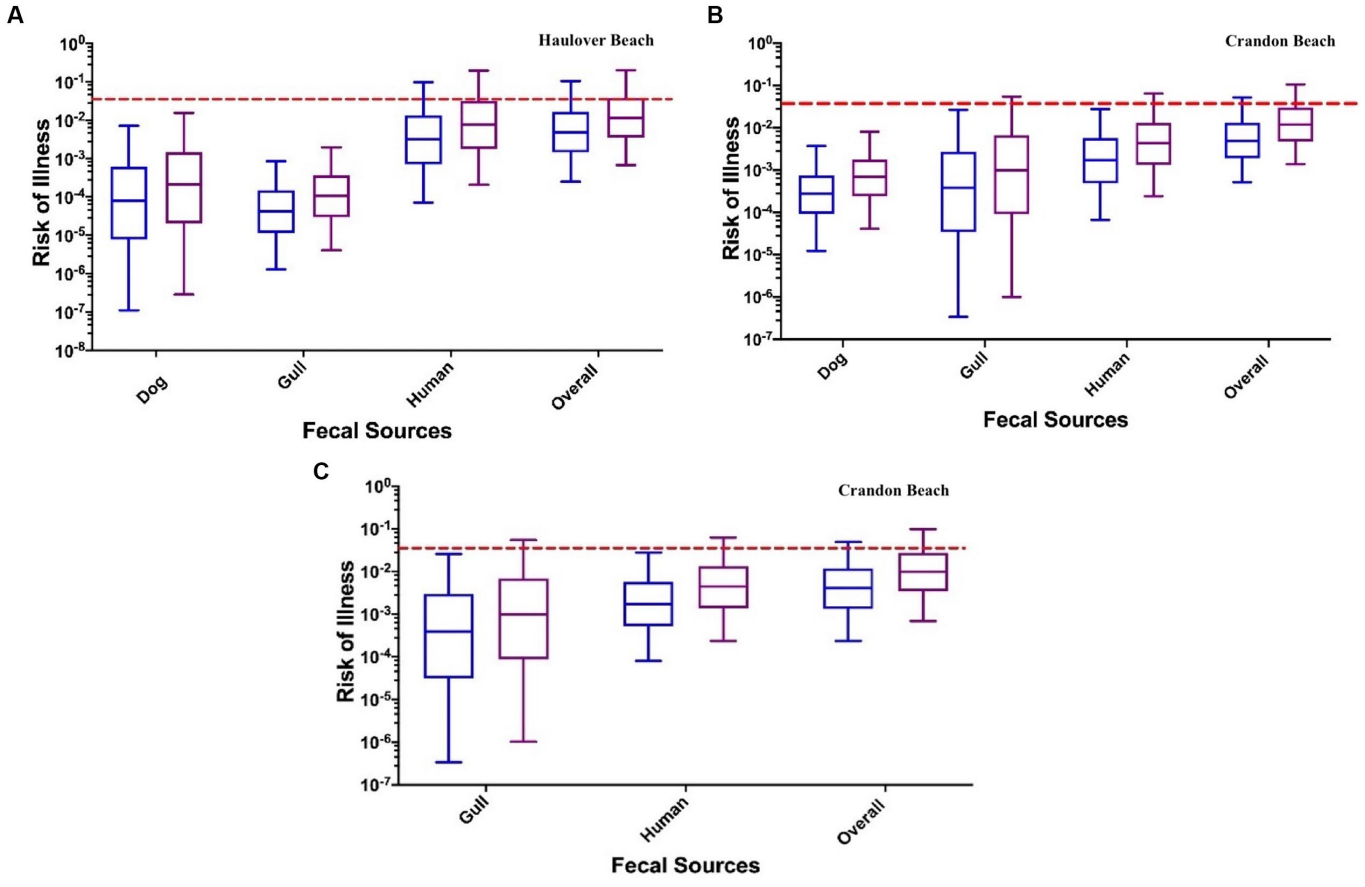
The risk of a GI illness per each fecal source: **(A)** Haulover Beach using the INT method for marker concentrations; **(B)** Crandon Beach assuming a DogBact concentration of 25 copies/100 mL (HF183 and Gull2 INT method for marker concentrations); **(C)** Crandon Beach assuming the dog fecal source is absent (HF183 and Gull2 INT method for marker concentrations). The left blue boxplots in each pair of results represent adults, and the right purple boxplots represent children. The dashed red line indicates the U.S. EPA risk threshold of 0.032.

At both CPB and HB, the human fecal source was identified to pose the greatest human health risk (Figure 5; Supplementary Figures S7–S9; Table 2). Health risks posed by human sources were an order of magnitude greater than the risks estimated for the dog and gull fecal markers. Median health risks from dog and gull fecal sources were comparable. For HB, the median health risks (from the INT data) associated with human sewage (3.15 × 10^−3^ for adults and 7.73 × 10^−3^ for children) were approximately one to two orders of magnitude higher than for the median health risks for dog (7.96 ×10^−5^ for adults and 2.10 × 10^−4^ for children) and gull (4.19 × 10^−5^ for adults and 1.08 × 10^−4^ for children) (Figure 5).

**Table 2:** Median probability of illness for both adults and children (adult | children) per each fecal source at both Haulover and Crandon Park Beaches.

Beach	Fecal source	Median risk of illness (INT method)	Median risk of illness (DL method)
Haulover	Dog	7.96 × 10^−5^|2.10 × 10^−4^	4.03 × 10^−4^| 1.01 × 10^−3^
	Gull	4.19 × 10^−5^| 1.08 × 10^−4^	6.69 × 10^−5^| 1.70×10^−4^
	Human	3.15×10^−3^|7.73 × 10^−3^	3.40 × 10^−3^|8.47 × 10^−3^
	Overall	4.75 × 10^−3^| 1.14 × 10^−2^	5.73 × 10^−3^|1.38 × 10^−2^
Crandon	Dog	2.78 × 10^−4^| 6.93 × 10^−4^	2.77 × 10^−4^| 6.73 × 10^−4^
	Gull	3.83 × 10^−4^|9.90 × 10^−4^	5.51 × 10^−4^| 1.40 × 10^−3^
	Human	1.70 × 10^−3^| 4.31 × 10^−3^	1.95 × 10^−3^| 4.77 × 10^−3^
	Overall	4.90 × 10^−3^|1.19 × 10^−2^	5.47 × 10^−3^| 1.30 × 10^−2^
	Overall (excluding Dog)	4.07×10^−3^| 9.95 × 10^−3^	4.45 × 10^−3^| 1.07 × 10^−2^

Alternatively, the health risk (from the DL data) for the dog fecal source (4.03 × 10^−4^ for adults and 1.01 × 10^−3^ for children) was slightly greater by one order of magnitude than the health risks estimated for the gull source (6.69 × 10^−4^ for adult and 1.70 × 10^−4^ for children) (Supplementary Figure S7). The overall human health risks for both data methods were approximately 1 × 10^−3^ for adults and 1 × 10^−2^ for children.

For CPB, the DogBact MST marker was not detected during any of the sampling events. The health risks from human sewage (1.70 × 10^−3^ for adults and 4.31 × 10^−3^ for children for INT data, 1.95 × 10^−3^ for adults and 4.77 × 10^−3^ for children for DL data) exceeded the non-human fecal sources (Figure 5; Supplementary Figures S8, S9; Table 2). Among the two scenarios which either did or did not include the dog fecal source, the estimated overall median human health risk was 1 × 10^−3^ for adults and ranged between 1 × 10^−2^ and 1 × 10^−3^ for children. When assuming no dog marker was present (and therefore absent of dog fecal contamination), the median health risks from the gull source was still one order of magnitude lower than the human source (INT data) (Figure 5; Supplementary Figure S8). When using the DL method for the data, the median risk of illness from the gull source (5.51 × 10^−4^ for adults and 1.40 × 10^−3^ for children) was either one order of magnitude lower or in the same order of magnitude as the median health risk from the human source (1.95 × 10^−3^ for adults and 4.77 × 10^−3^ for children) (Supplementary Figure S8). When including the dog fecal source, the median health risks for dog (2.78 × 10^−4^ for adults and 6.93 × 10^−4^ for children) were within the same order of magnitude as the gull fecal source (3.83 × 10^−4^ for adults and 9.90 × 10^−4^ for children) for the INT data (Figure 5).

When exposed to a mixture of fecal sources at both CPB and HB, the median human health risks were all below the U.S. EPA risk threshold of 0.032 (Figure 5; Supplementary Figures S7–S9). A slight difference in health risks were evident between adults and children, which is likely due to the assumed greater ingestion volume of marine water for children compared to adults when swimming (DeFlorio-Barker et al., 2018). While the data when fitted to distributions utilizing INT and DL methods yielded similar overall human health risks, the median risks associated with each fecal source did vary. The health risks from the human fecal source appears to drive the overall health risk when exposed to a mixture of fecal sources.

#### 3.2.2. Risks associated with reference pathogens

**Figure 6:**
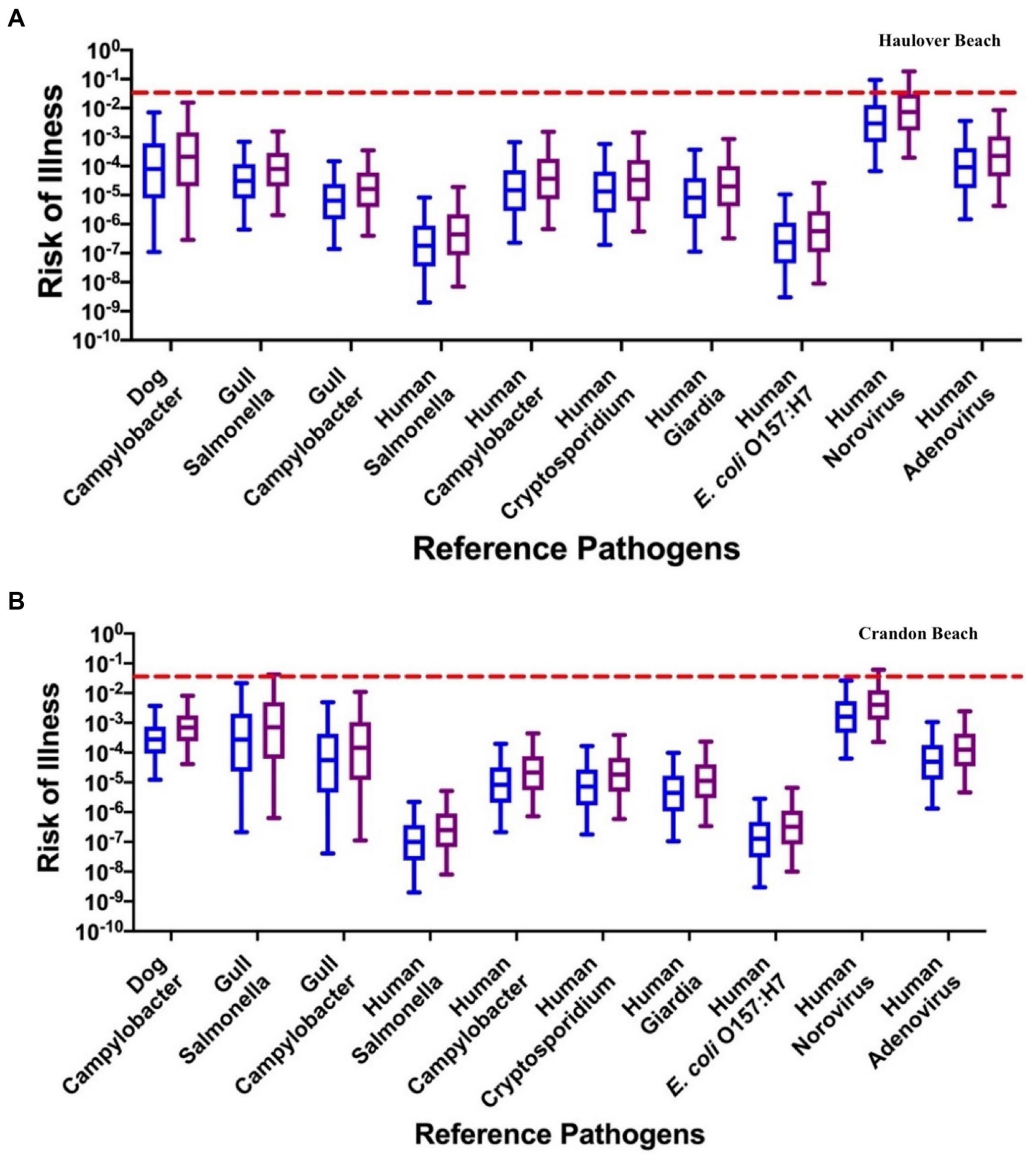
The risk of illness associated with each reference pathogen: **(A)** Haulover Beach using the INT data for HF183, Gull2 and DogBact MST markers; **(B)** Crandon Beach assuming a concentration of 25 copies/100 mL for the DogBact MST marker and INT data for HF183 and Gull2 MST markers. The left blue boxplots represent adults, and the right purple boxplots represent children. The dashed red line indicates the U.S. EPA risk threshold of 0.032.

For HB, the median risks of illness from *Campylobacter* from the dog fecal source (7.96 × 10^−5^ for adults and 2.10 × 10^−4^ for children) were estimated to be slightly greater than the median health risks posed by *Campylobacter* in gull feces (6.41 × 10^−6^ for adults and 1.62 × 10^−5^ for children); however, these health risks were similar to the median health risks posed by *Campylobacter* in human sewage (1.49 × 10^−5^ for adults and 3.65 × 10^−5^ for children) for the INT data (Figure 6; Supplementary Table S4). For gulls, the human health risks for a GI illness associated with *Salmonella* (6.51 × 10^−7^ for adults and 7.96 × 10^−5^ for children) were lower than the risks from *Campylobacter*. Norovirus had the greatest median health risk for the human source (2.97 × 10^−3^ for adults and 7.34 × 10^−3^ for children) and has been identified to dominate the health risk in other recreational and drinking water studies (Stampi et al., 1993; Schoen and Ashbolt, 2010; Hunter et al., 2011; Ervin et al., 2014; Van Abel et al., 2017a). Adenovirus had the second greatest median health risk for the human source (9.22 × 10^−5^ for adults and 2.24 × 10^−4^ for children), while *Salmonella* (2.00 × 10^−9^ for adults and 4.47 × 10^−7^ for children) and *E. coli* O157:H7 (3.00 × 10^−9^ for adults and 5.75 × 10^−7^ for children) had the lowest median health risks. The health risks associated with adenovirus were within the same order of magnitude as the health risks associated with the *Campylobacter* reference pathogen for the dog source. The median health risks for each pathogen utilizing the DL method were within an order of magnitude as the estimated health risks using the INT method (Figure 6; Supplementary Figure S10; Supplementary Table S4).

For CPB, when assuming the dog fecal source was present, the relative human health risks were similar to those identified with HB (Figure 6; Supplementary Figures S10, S11; Supplementary Table S4). The risk of illness for norovirus (1.61 × 10^−3^ for adults and 4.06 × 10^−3^ for children) was again the reference pathogen with the greatest median risk under the INT data method. However, the median health risks from *Campylobacter* for dog (2.78 × 10^−4^ for adults and 6.93 × 10^−4^ for children) and gulls (5.55 × 10^−5^ for adults and 1.44 × 10^−4^ for children) and *Salmonella* for gulls (2.74 × 10^−4^ for adults and 7.07 × 10^−4^ for children) were within the same order of magnitude as the median health risks associated with adenovirus (4.92 × 10^−5^ for adults and 1.25 × 10^−4^ for children). For the human reference pathogens, both *Salmonella* (1.00 × 10^−7^ for adults and 2.49 × 10^−7^ for children) and *E. coli* O157:H7 (1.27 × 10^−7^ for adults and 3.23 × 10^−7^ for children) had the lowest median health risks, similar to HB. The risk of illness for each reference pathogen for both the INT and DL methods, and for when the dog fecal source was absent, were all within the same order of magnitude (Figure 6; Supplementary Figures S11–S13; Supplementary Table S4).

#### 3.2.3. Sensitivity analysis

A sensitivity analysis of the human health risks associated with all fecal sources was conducted for the input parameters that were defined by distributions (Supplementary Table S3) using the rank correlation approach (Supplementary Figures S14–S25). Generally, the model was identified to be most sensitive to the concentration of the HF183 marker in seawater. For HB, the QMRA model was also sensitive to the adult and child ingestion rates and the DogBact MST marker concentration in the environment. However, for the CPB models, the Gull2 marker in seawater and adult and child ingestion rates were identified as being the second and third most sensitive parameters. Among all simulations for both beaches, the input parameters describing the concentrations of pathogens and MST markers in different fecal sources (e.g., in human sewage, gull or dog feces) did not appear to have as great of an influence on the risk output. The consistency of HF183 being identified as the most sensitive parameter emphasizes that the sources of the human fecal marker are primary drivers of the GI illness risk from these exposures.

## 4. Discussion

Minor differences in beach structure, tidal influences, sand composition, and local weather are well documented to potentially impact the microbial landscape beyond the influence of humans (Whitman et al., 2014). When combined with anthropogenic impacts, such as sewer/septic leaks, contaminated storm water runoff, and other sources of land-based pollution, it can be difficult to differentiate the sources that truly represent a public health concern for recreational bathers. While traditional methodologies such as enterococci plate counts serve as the primary method for beach screenings, their limitations are well known. MST allows for a more targeted understanding of the different microbial contaminants reaching coastal sites and beaches, yet it may still be difficult to independently determine potential health risks for bathers, especially since exposure thresholds and beach advisory guidelines have not been established for most MST markers.

The low or absent levels of dog marker observed during this study is not unexpected, as dogs are not permitted on either beach, although there is an established dog run near HB. Water samples from HB had the only detects of DogBact above the LLOQ (3 samples) (see Supplementary material for more details about the LLOQ). The low levels of dog fecal marker observed at HB, despite the close proximity of a dog run, suggests relatively effective enforcement of dog hygiene and cleanup practices at this site.

Based on the QMRA simulations presented in this study, the GI illness risk from swimming or playing in water containing a mixture of human and non-human fecal sources appear to be primarily driven by the human fecal source. However, the estimated median GI health risk for both CPB and HB never exceeded the U.S. EPA risk threshold. Thus, identifying which reference pathogens and fecal sources have the greatest influence on risk is imperative for effective beach management and protecting public health. While the human source did appear to “drive” the overall health risk, both the dog and seagull fecal sources had comparable estimated health risks.

The gull source was detected more frequently than the dog source at both beaches. However, the dog fecal source, while not detected at as high of concentrations as the gull source, still presented a potential health risk in these QMRA simulations. While gull management may be challenging to implement for beaches, at least limiting dogs on or upstream of recreational beaches (and/or implementing a robust and enforced pet waste cleanup policy) can not only reduce potential fecal loads into the water body, but also lessen any potential health risks. Gull feces, while apparently not as great of risk to health as human fecal contamination, should still not be disregarded for beach management and public health. Attempts at curtailing gull presence at some beaches have been implemented with successful reduction of FIB and pathogen densities (Converse et al., 2012).

The human fecal source for this QMRA estimation was represented by sewage, in which the human HF183 marker in sewage is predicted to have a greater median health risk than when detected in treated effluent (Brown et al., 2017b). CPB and HB were not determined to have any permitted wastewater outfalls. In fact a substantial component of the HF183 marker may have derived from bather shedding (Li et al., 2021); therefore assuming human sewage as a “worst case” source was determined to be more appropriate for this study. This assumption of sewage as the HF183 marker source provided a more conservative and protective approach for evaluating the specific human health risks of exposure leading to a GI illness at both beaches. However, it should be recognized that there are still other significant non-GI health endpoints for which the assumption of sewage as the human fecal marker source may not be as relevant or as protective. The frequent detection of HF183 in daily samples at both beaches (despite lacking any known nearby sources of sewage exposure) also indicates that this human fecal marker likely results from bather shedding, which while possibly providing a greater risk for exposure to skin pathogens such as *Staphylococcus aureus,* would still presumably be a lesser risk than for enteric pathogens.

Exposure parameters for both adults and children were retrieved from a study which pooled 12 prospective cohorts (approximately 68,685 participants) to examine exposure durations, frequency, and ingestion volumes when swimming and recreating in water (DeFlorio-Barker et al., 2018). However, a future QMRA should evaluate how site-specific exposure behaviors, as documented in Ferguson et al. (2021a) influence these human health risk estimates. Other exposure routes, including the incidental ingestion of water through wading, fishing, water skiing, and kayaking could also be of health concern (Dorevitch, 2011). While health risks associated with seawater ingestion were evaluated, both sand and seaweed wrack could pose significant health concerns for recreators. Beach sand has been identified as an exposure pathway for a variety of different pathogens, emphasizing the importance of incorporating sand sampling and pathogen enumeration into regulatory programs (Solo-Gabriele et al., 2016). Future research should strive to evaluate human health risks associated with exposure to microbial contaminants, as indicated by MST gene markers, in both of these media, given that a significant portion of time at the beach is spent on the shore and not in water.

The health risks estimated in this study complement previous microbial risk assessments which evaluated health risks associated with recreational waters, in light of different fecal pollutants. McGinnis et al. (2022) identified an increase in health risks (acute gastrointestinal illnesses) associated with recreation in urban waterways in Philadelphia in the 24 h following a sewer overflow event (contamination from a human fecal source). Previous work by Boehm et al. (2015, 2018) and Boehm and Soller (2020) have proposed RBTs for specific MST markers for fecal sources frequently detected in recreational waters (HF183 and gull). Ahmed et al. (2018) suggested HF183 RBTs that varied depending on if contamination was from fresh sewage or secondary treated sewage. Findings in our work are supported by these previous studies in that our measurements of the MST markers did not exceed the proposed RBTs and also estimated health risks did not exceed the 0.032 recreational water quality risk threshold. Additionally, the health risks derived from the HF183 marker, specifically for the reference pathogen norovirus, contributed the majority of the risk across all the integrated QMRA-MST studies.

The sensitivity analysis indicated that the MST marker concentration, specifically for HF183 and Gull2, had the greatest influence on the QMRA model. Managing the specific fecal sources, such as the human source, will likely have the greatest impact on reducing risk. Although exposure typically drives microbial infection risks, this study demonstrates the importance of source identification when addressing bather health risks in recreational waters. Therefore, beach management should continue to target minimizing contamination from human and non-human fecal sources, if possible, given those are primary factors influencing risk.

Lastly, a revised RBT for fecal contamination of unknown age was determined in a previous QMRA study to be 525 copies/100 mL for HF183 and 200,000 copies/100 mL for gull feces (Boehm and Soller, 2020). The study we present here assumes fresh contamination of sewage, gull, and dog feces (three contributing fecal sources), which could indicate why the overall median risk of illness may be only one order of magnitude less than the risk threshold of 0.032. The risk of illness outcomes estimated in this study do align with the previously published RBTs. Applying these proposed RBTs in a real-world context is not only informative for evaluating site-specific recreational water quality, but useful in assessing the appropriateness of these thresholds for beach management.

### 4.1. Study limitations

Certain assumptions and limitations in the study design and QMRA may have had an impact on the overall risk output. It was assumed that the fecal sources, human sewage, dog, and gull feces, were all fresh with no aging, which is a similar approach used in other QMRAs (Soller et al., 2010; Brown et al., 2017b). Other recent QMRAs have incorporated fecal aging but have indicated that fecal sources are likely composed of a mixture of ages and overall risk estimates may be sensitive to the decay rate constants used for certain pathogens, such as norovirus. Future risk assessments utilizing environmental data could be refined to include pathogen and MST marker decay, and those ratios of decay may influence health risk outcomes. Refining approaches of risk analyses that are conservative and have incorporated unknown and mixed ages of fecal sources could help develop a robust risk simulation for beach managers. However, the approach presented in this study provides a conservative risk estimate and protection for human health.

This QMRA study relied upon input parameters (as described in Supplementary Table S3) and dose–response relationships (Table 1) gathered from the literature. The pathogen and MST concentrations, ingestion volumes and range of morbidity for pathogens, likely vary among different environments. Assumptions must be made to incorporate these values into the risk assessment and are assumed to be the best available information at this time. Utilizing MST markers for site-specific risk assessments is an advancement in recreational water quality monitoring, but there are limitations associated with these markers. MST markers are not 100% host specific and sensitive, such as with the Gull2 marker, which has displayed limited cross-reactivity with other seabird species, as well as some pigeon populations. However, multiple species of seabirds and other birds (such as certain terns, geese, or pigeon populations) may also carry the same *C. marimammalium* species that is targeted by the Gull MST assay as normal intestinal flora. Consequently, these other species of birds may represent actual proper target detection as opposed to marker specificity cross reactivity. Given similar co-nesting and scavenging behavior with gulls by these other birds also carrying intestinal *C. marimammalium* (such as pigeons), the feces from these other birds which test positive for the Gull MST fecal marker may likely pose a similar risk as gull feces.

Due to the small sample size, the results of this study are considered preliminary and can be used to guide further exploration. Application of these results by health and regulatory authorities will require testing at additional sites, and benchmarking against current culture-based methods currently used to assess recreational water quality. In addition, water samples were collected at one depth during a one-week time frame, therefore only capturing a snapshot of environmental conditions occurring at both beaches. Future studies should evaluate multiple sampling depths and conduct more frequent sampling. Some precipitation did occur during sampling and could have elevated the concentrations of these MST fecal markers, potentially increasing human health risks associated with swimming. Future work evaluating health risks pre – and post – precipitation would be valuable for identifying not only potential human health risks, but also identifying the predominant fecal sources in stormwater run-off.

Despite these various limitations, assessing health risks associated with MST markers – as opposed to FIB concentrations – provides greater insight into the variety of fecal sources impacting a water body, and therefore better informing targeted application of best management practices. Personnel with skilled training, laboratory infrastructure and funding are necessary for this approach of utilizing molecular markers for beach monitoring. While qPCR methods have been approved by the U.S. EPA for same-day decision-making for beach management, there are very few agencies and/or communities implementing this approach. While rapid beach water testing has the potential to provide timely results for public health consideration (within the day), less than 1% of beach monitoring is currently conducted with rapid methods (Shrestha and Dorevitch, 2020). Laboratories employing qPCR methods, instead of culture-based methods, do incur additional costs [according to 2015–2017 estimated dollars, equipment supplies would cost about USD $73,000 (Shrestha and Dorevitch, 2020)], yet cost-savings from mitigating gastrointestinal illnesses (due to rapid detection methods) should not be overlooked. In addition, the development of standard reference materials by the National Institute of Standards and Technology and U.S. EPA, to ensure accuracy of water quality methods and results for microbiological labs conducting water quality testing, is a key step in implementing qPCR approaches for beach management on a larger scale (Boss, 2022). While these are limitations, if molecular data or the resources are available for site-specific MST, this approach should be pursued.

As indicated in this study, the GI risk from swimming or playing in water containing a mixture of human and non-human fecal sources will be driven by the human fecal source. Library-independent MST fecal markers, specifically HF183, Gull2, and DogBact, were used to represent the potential fecal contamination from sewage, gull, and dog feces. The approach used in the current study, albeit a conservative method for assessing risk at two popular recreational beaches, is an application of using MST markers to evaluate the GI risk associated with swimming or other contact activities, while utilizing a methodology that applies a ratio of MST markers in the environment and in sewage/feces to estimate pathogen concentrations (Brown et al., 2017b; Boehm and Soller, 2020). In order to assess risks more broadly for beach recreational use, MST should be integrated into assessments that also evaluate risks from contact with sand and seaweed.

## 5. Conclusion

This risk assessment is a case-study applied approach of utilizing environmental MST marker data for different fecal sources at popular recreational beaches in a QMRA. This study is a first attempt at evaluating the proposed HF183 and Gull2 marker RBTs (Boehm and Soller, 2020) for beach management decision-making. This QMRA study can serve as a starting point for beach managers to assess health risks from not only human sewage and gull feces, but also dog feces. While the detection of traditional FIB (specifically enterococci) has been useful for managing water quality, limitations of live FIB enumeration such as with environmental regrowth and persistence have posed challenges for adequately assessing recreational water quality and safety. Our research suggests that incorporating a QMRA approach along with other methodologies, specifically MST, could be of benefit for recreational beaches where more traditional methodologies, such as enterococci plate counts for detection of FIB, may have previously given mixed or inconclusive results. Utilizing QMRA may, in certain situations, further extend our calculations and understanding of potential recreational bather risk. Future studies should include traditional measurements of FIB along with MST measurements. Applied approaches of utilizing site-specific environmental MST data in QMRA studies that can be developed not only by public health practitioners, but also by beach managers, will ultimately help direct budgeted resources to be used effectively and implement management strategies that support public health. The benefit of targeting specific fecal sources in risk analyses for beach management should not be overlooked, given its ability to determine when and where it is safe for beach recreation.

## Data availability statement

The raw data supporting the conclusions of this article will be made available by the authors, without undue reservation.

## Author contributions

AG conceived and designed the QMRA analysis, conducted all QMRA analysis, contributed analysis tools, analyzed data, prepared figures and tables, authored, and reviewed drafts of the manuscript. MG conceived and designed the experiments, performed the experiments, contributed reagents, materials, analysis tools, analyzed data, prepared figures and tables, authored, and reviewed drafts of manuscript. KDM assisted in the design of the QMRA analysis, contributed analysis tools, analyzed data, and reviewed drafts of manuscript. AF was responsible for the collection of video data and activity patterns assessments for the overall BEACHES project, and reviewed drafts of the manuscript. CS conceived and designed the experiments, performed the experiments, oversaw, and coordinated all microbiological analysis and molecular microbial source tracking analysis, contributed reagents, materials, analysis tools, analyzed data, prepared figures and tables, authored, and reviewed drafts of manuscript. AB conducted the primary qPCR microbial source tracking analysis, collected and processed field samples, conducted eDNA extraction and purification, performed the experiments, and reviewed drafts of the manuscript. HS-G conceived and designed the experiments and the overall BEACHES project, oversaw all aspects of the project, helped with project field work, and reviewed drafts of the manuscript. All authors contributed to the article and approved the submitted version.

## Author disclaimer

Neither the United States Government nor any of its employees, contractors or their employees make any warranty, expressed or implied, or assumes any legal liability or responsibility for any third party’s use of apparatus, product, or process discussed in this document, or represents that its use by such party would not infringe on privately owned rights. Mention of any commercial entities, trade names, or commercial products in this document does not constitute any type of endorsement or recommendation for use. The scientific results and conclusions, as well as any views or opinions expressed herein, are those of the author(s) and do not necessarily reflect those of NOAA, OAR, or the Department of Commerce.
