# Supplementary material for: Integrating microbial source tracking with quantitative microbial risk assessment to evaluate site specific risk based thresholds at two South Florida beaches
Source: Front Microbiol. 2023 Oct 12;14:1210192. doi: 10.3389/fmicb.2023.1210192 (PMC10602684; doi:10.3389/fmicb.2023.1210192)
Supplement: Supplementary file 1 [file Data_Sheet_1.pdf]

Supplementary Material

Integrating Microbial Source Tracking with Quantitative Microbial Risk Assessment to Evaluate Site Specific Risk Based Thresholds at Two South Florida Beaches

Anna Gitter[1*], Maribeth Gidley[2,3], Kristina D. Mena[1], Alesia C. Ferguson[4], Christopher Sinigalliano[2], Anthony Bonacolta[5,6], Helena Solo-Gabriele[6]

*** Correspondence:** Anna Gitter: anna.gitter@uth.tmc.edu

1 Supplementary Details on Materials and Methods

1.1 Study Area and Study Design

Sand and seaweed wrack were aseptically collected, along with the water samples, at CPB and HB during the BEACHES child beach exposure study. Both CPB and HB were sampled on 4 consecutive days at 3 time points each day (morning before the start of the child observations, mid-day during the child observations, and late afternoon following the conclusion of the child observations) during the period of June 21st, 2018 to June 24th, 2018 and June 27th, 2018 to June 30th, 2018, respectively.

1.2 Sample Collection and Preservation

Water: See methods section of main article text.

Sand: Samples of dry sand were collected from just above the high-tide wrack line, while samples of wet sand were collected from the intertidal swash zone. For each sand sample, approximately 40cc of sand from the top 3-5cm was aseptically transferred into a sterile 50mL polypropylene centrifuge tube using a fresh sterilized spoon for each sample transfer. All sand sample tubes were immediately stored on ice following collection and transported back to the laboratory on ice within 6 hours of collection for further sample processing and preservation. At the lab, the 40cc sand sample was decanted into a large sterile polypropylene weight boat and thoroughly mixed with a sterile spatula, after which a 10g sub-aliquot of this homogenized sand was aseptically transferred to a sterile 1L Whirlpak bag (NASCO) along with 500mL of sterile 1X Phosphate Buffered Saline (PBS) and sealed. Bacteria were resuspended from the sand into the 1X PBS by vigorous shaking and agitation by hand, including rubbing and kneading, for 10 minutes, followed by 10 minutes of settling on the benchtop. After settling, 100mL of the bacterial PBS resuspension was aseptically filtered onto 0.45 µm pore-sized mixed cellulose ester 47mm diameter filters (GN-6 Metricel, Pall Corp), which were aseptically rolled and transferred into sterile 2mL polypropylene tubes. Filters were then stored at -80°C until extraction.

Seaweed Wrack: Samples of dried seaweed wrack from the high tide wrack line (consisting predominantly of *Thalassia* and *Sargassum* spp.) was collected aseptically by hand and transferred into sterile Whirlpak bags (NASCO), by samplers wearing nitrile gloves that had been sanitized with 70% ethanol spray after donning gloves and dried before wrack collection. All seaweed sample bags

were immediately stored and transported back to the laboratory on ice within 6 hours of collection for further sample processing and preservation. At the lab, 0.5g sub-samples were harvested from the sample bags with sterile forceps, weighed aseptically, and transferred to sterile “Lysing Matrix A” bead beat homogenization tubes (FastDNA Spin Kit, MPBiomedicals), then stored at -80°C until extraction.

1.3 Extraction and Purification of Environmental DNA

For samples from water or sand resuspensions, the frozen filter was aseptically transferred to a “Lysing Matrix E” bead-beat homogenization tube from the FastDNA Spin Kit for Soil (MPBiomedicals) along with 1mL of lysis buffer from the kit as per manufacturer’s instructions, then the sample was homogenized by 2 rounds each of bead-beating in a FastPrep-24 homogenization instrument (MPBiomedicals) with an impact speed setting of 6.0 m/s for 60 sec each (with a 5 minute cool-down period between each round of bead-beating). Before homogenization, the lysis buffer was amended with Chum Salmon DNA at 0.2ug/mL as a Sample Processing Control (SPC, as per EPA Method 1696). The lysate tubes were centrifuged at >14,000 x g for 15 minutes to pellet debris, then the lysate was transferred and purified using the FastDNA Spin Kit for Soil as per manufacturer instructions and eluted in a final volume of 100uL with the kit’s elution buffer. The eluted purified eDNA samples were divided into replicate aliquots and stored frozen at -20°C until qPCR analysis.

For frozen seagrass wrack samples, the 0.5g wrack samples in the “Lysing Matrix A” tubes were directly extracted by bead-beating homogenization in the tube using the FastDNA Spin Kit with 1 mL of the kit’s CLS-VF plant lysis buffer (amended with Chum Salmon DNA at 0.2 ug/mL) in a FastPrep-24 homogenization instrument (MPBiomedicals), using 2 rounds of homogenization with an impact speed setting of 6.0m/s for 60 sec (with a 5 minute cool-down period between each round of bead-beating). Tubes were centrifuged at >14,000xg for 15 minutes to pellet debris, then the lysate was transferred and purified using the FastDNA Spin Kit as per manufacturer instructions and eluted in a final volume of 100uL with the kit’s elution buffer. The eluted purified eDNA samples were divided into replicate aliquots and stored frozen at -20°C until qPCR analysis.

1.4 MST qPCR Analysis

Modifications to the MST qPCR analyses are as follows:

In the case of the enterococcus Entero1A assay, this was quantitated using standard concentration curves of known concentrations of purified genomic DNA from *Enterococcus faecalis* (strain ATCC-29212) following the standard curve protocols and qPCR QA/QC metrics of EPA methods 1696 and 1697, rather than using the calibrator cell equivalent (cce) protocol of EPA method 1611. For all qPCR assays, protocols were slightly modified from the reference source as follows: all assays were conducted in 25 µL PCR reaction volumes on a StepOnePlus Real-Time PCR system (Applied Biosystems) as simplex reactions, therefore for each reaction cocktail: 12 µL of Qiagen QuantiTect Probe PCR mastermix (Qiagen), 2 µL DNA template (either unknown sample, or quantitation standard, or no template negative control respectively), 7.5 µL of PCR-grade water, and 3 µL of the corresponding primers-probe mix specific for each assay. Final concentrations for the forward and reverse primers were 1 µM and final concentrations of the 5’-exonuclease probes were 80 nM. Cycling conditions were 15 minutes at 95°C to activate the hotstart polymerase enzyme of the mastermix, followed by 40 repetitive cycles of the 95°C denaturation step for 15 sec, followed by the 60°C annealing and extension step for 1 minute, with a fluorescence reading of each well at the end

of each extension step. The baseline and fluorescence cycle threshold (CT) were set to automatic by the instrument. The sequences and characteristics of the primers, probes, and positive control standards used in this study are summarized in Supplementary Table S-1. Consensus triplicate standard curves for each assay were used to convert Cq values (the fraction of cycles for each reaction where the amount of fluorescence reaches the set cycle threshold in the log phase of the amplification) into the calculated copy number of sequence targets per well (designated here as "Target Sequence Copies" or "TSC").

The positive control target DNA concentration standards were constructed from dilutions of a known copy number of synthetic double-stranded target DNA standards ("gBlocks" synthetic gene fragments, synthesized by Integrated DNA Technologies), ranging from 10^5 to 10^1 gene copies per reaction well for the standards. In the case of the general enterococcus entero1A assay, dilutions of a known genome copy (gc) number of the purified *Enterococcus faecalis* genomic DNA were used instead of synthetic gene blocks of the target sequence. The quantities in target sequence copy number (or genome copy number in the case of enterococci) for the environmental samples were then determined by comparing their Cq values to the averaged positive control standard curve for that assay batch to determine the copies per reaction, then adjusted for the sample filtration, dilution, and elution volumes to give the final copies per 100 mL of water sample or per gram dry weight of sediment or seaweed wrack.

qPCR QA/QC: The U.S EPA describes quality assurance procedures and controls in Method 1696 (2019) for the characterization of human fecal pollution in water by HF183. These quality control metrics and guidelines were also used in this study for all qPCR markers as well. Master Standard Curves were created for each of the sets of replicate curves for each assay and used for the evaluation of compliance to the QA/QC metrics, including the determination of the Lower Limit of Quantitation (LLOQ) for each assay. Specifics of these QA/QC metrics for the qPCR assays used in this study are listed in Table S2 below. Negative qPCR controls consisted of "NTC" or no template controls, which is where no target DNA was added to the reaction wells. A determination of "Not Detected" (ND) was made whenever the level of target gene amplification of a sample was not sufficient for the fluorescence signal to be able to reach the level of the Cycle Threshold (CT). Positive detection is defined as the increase in fluorescence signal from the targeted gene amplification to the point where this fluorescence reaches the level of the CT value. The measured Cq value of positive detections is the fraction of the cycle where the amplification reaches the level of this Cycle Threshold. The Limit of Detection (LOD) for each assay was judged to be 1 copy per reaction based upon the frequent but not reliably repeatable detection of the 1 copy/reaction concentration standards. The 10 copy per reaction concentration standard of each assay was judged to be the lowest reliable and repeatable detected standard. This $1\log_{10}$ standard was therefore used for determining the LLOQ of the reaction as per EPA Method 1696. The range between the LOD and the LLOQ is designated as the "Detected but Not Quantified" or "DNQ" range, and target concentration estimates in this DNQ range should be considered as uncertain and/or insignificant since they are below the threshold of reliable quantitation under these particular conditions of sample processing and qPCR reaction. As per EPA Method 1696, the lower limit of quantitation (LLOQ) per reaction was determined from the master standard curves of each gene target, where the Cq of the reaction LLOQ was represented by the Cq of 95% prediction upper limit of the 1 $\log_{10}$ copy of the control target DNA standard dilution. This LLOQ Cq value was used to measure the LLOQ in copies/reaction, based upon the equation of the standard curve linear regression (see Table S2 for slope and Y-intercept of each assay consensus regression equation). The environmental LLOQ (the "eLLOQ") was then calculated based upon the volume of original environmental sample that was extracted, the amount of lysis buffer extracted into, the

various dilutions made during the extraction/purification process, the final purified eDNA elution volume of each extraction, and the volume of this eluate that was used in the qPCR reactions (2 uL of sample DNA for each reaction). All of the individual assay LLOQs were close to and ≤ 10 copies/reaction (see Table S2 for each specific assay's LLOQ), so for purposes of calculating a general environmental LLOQ and for graphing and visualization purposes, a standardized LLOQ of 10 copies/reaction was selected for each assay. This represents a conservative exposure estimate of the LLOQ as compared to the actual measured LLOQ of any individual assay. This does give a slightly higher range of DNQ for most of the assays as compared to the individual assay's measured LLOQ (this admittedly may cause some slight decrease in the sensitivity of the threshold of reliable quantitation for low concentrations thus giving a slightly larger DNQ range), but this allows for easier graphic and visualization comparisons of samples higher than the DNQ range across assay types for a particular environmental matrix (i.e. either water, sand, or seaweed wrack).

The quality control parameters described here represent the average of all the replicate standard curves for a particular assay. "E" is the amplification efficiency, calculated as "E = -1 + 10^(-1/slope)". The E value should typically be between 0.90 and 1.10. All run standard curves for all markers had an $R^2 \geq 0.98$, E values were between 90% and 110%, NTCs were all non-detects, and the Cq values of the Sketa22 assay for the Salmon Chum Sample Processing Controls (SPC) were within 3 standard deviations of the method blanks, so all other Quality Control parameters outlined in EPA Method 1696 were satisfied.

<u>Literature Cited in Supplement:</u>

- Brown, K. I., Graham, K. E., and Boehm, A. B. (2017a). Risk-Based Threshold of Gull-Associated 625 Fecal Marker Concentrations for Recreational Water. *Environ. Sci. Technol. Lett.* 4, 44–48. 626 doi:10.1021/acs.estlett.6b00473.

- Chaban, B., Ngeleka, M., and Hill, J. E. (2010). Detection and quantification of 14 Campylobacter species in pet dogs reveals an increase in species richness in feces of diarrheic animals. *BMC Microbiol.* 10. Available at: http://www.biomedcentral.com/1471-2180/10/73.

- Crockett, C. S. (2007). The Role of Wastewater Treatment in Protecting Water Supplies Against Emerging Pathogens. *Water Environment Research* 79, 221–232. doi: 10.2175/106143006X111952.

- DeFlorio-Barker, S., Arnold, B. F., Sams, E. A., Dufour, A. P., Colford, J. M., Weisberg, S. B., et al. (2018). Child environmental exposures to water and sand at the beach: Findings from studies of over 68,000 subjects at 12 beaches. *J. Expo. Sci. Environ. Epidemiol.* 28, 93–100. doi:10.1038/jes.2017.23.

- Eftim, S. E., Hong, T., Soller, J., Boehm, A., Warren, I., Ichida, A., et al. (2017). Occurrence of norovirus in raw sewage – A systematic literature review and meta-analysis. *Water Research* 111, 366–374. doi: 10.1016/j.watres.2017.01.017.

- Ervin, J. S., Van De Werfhorst, L. C., Murray, J. L. S., and Holden, P. A. (2014). Microbial source tracking in a coastal California watershed reveals canines as controllable sources of fecal contamination. *Environ. Sci. Technol.* 48, 9043–9052. doi:10.1021/es502173s

- García-Aljaro, C., Bonjoch, X., and Blanch, A. r. (2005). Combined use of an immunomagnetic separation method and immunoblotting for the enumeration and isolation of Escherichia coli O157 in wastewaters. *Journal of Applied Microbiology* 98, 589–597. doi: 10.1111/j.1365-2672.2004.02497.x.

- Gras, L. M., Smid, J. H., Wagenaar, J. A., Koene, M. G. J., Havelaar, A. H., Friesema, I. H. M., et al. (2013). Increased risk for Campylobacter jejuni and C. coli infection of pet origin in dog owners and evidence for genetic association between strains causing infection in humans and their pets. *Epidemiol. Infect.* 141, 2526–2535. doi:10.1017/S0950268813000356.

- Griffith JF, Layton BA, Boehm AB, Holden PA, Jay JA, Hagedorn C, McGee CD, Weisberg SB. 2013. The California Microbial Source Identification Manual: A Tiered Approach to Identifying Fecal Pollution Sources to Beaches. Southern California Coastal Water Research Project (SCCWRP), Technical Report 804, 83 pp. Available at https://www.waterboards.ca.gov/water_issues/programs/beaches/cbi_projects/docs/sipp_manual.pdf

- Harwood, V. J., Staley, C., Badgley, B. D., Borges, K., and Korajkic, A. (2014). Microbial source tracking markers for detection of fecal contamination in environmental waters: Relationships between pathogens and human health outcomes. *FEMS Microbiol. Rev.* 38, 1–40. doi:10.1111/1574-6976.12031.

- Hewitt, J., Leonard, M., Greening, G. E., and Lewis, G. D. (2011). Influence of wastewater treatment process and the population size on human virus profiles in wastewater. *Water Research* 45, 6267–6276. doi: 10.1016/j.watres.2011.09.029.

- Hurst, C. J., McClellan, K. A., and Benton, W. H. (1988). Comparison of cytopathogenicity, immunofluorescence and In situ DNA hybridization as methods for the detection of adenoviruses. *Water Research* 22, 1547–1552. doi: 10.1016/0043-1354(88)90167-4.

- Kitajima, M., Haramoto, E., Iker, B. C., and Gerba, C. P. (2014). Occurrence of Cryptosporidium, Giardia, and Cyclospora in influent and effluent water at wastewater treatment plants in Arizona. *Science of The Total Environment* 484, 129–136. doi: 10.1016/j.scitotenv.2014.03.036.

- Koivunen, J., Siitonen, A., and Heinonen-Tanski, H. (2003). Elimination of enteric bacteria in biological–chemical wastewater treatment and tertiary filtration units. *Water research* 37, 690–698.

- Lemarchand, K., and Lebaron, P. (2003). Occurrence of Salmonella spp. and Cryptosporidium spp. in a French coastal watershed: relationship with fecal indicators. *FEMS Microbiology Letters* 218, 203–209. doi: 10.1111/j.1574-6968.2003.tb11519.x.

- Lévesque, B., Brousseau, P., Bernier, F., Dewailly, É., and Joly, J. (2000). Study of the bacterial content of ring-billed gull droppings in relation to recreational water quality. *Water Research* 34, 1089–1096. doi: 10.1016/S0043-1354(99)00266-3.

- Nasser, A. M. (2015). Removal of Cryptosporidium by wastewater treatment processes: a review. *Journal of Water and Health* 14, 1–13. doi: 10.2166/wh.2015.131.

- Schoen, M. E., Ashbolt, N. J., Jahne, M. A., and Garland, J. (2017). Risk-based enteric pathogen reduction targets for non-potable and direct potable use of roof runoff, stormwater, and greywater. *Microb Risk Anal* 5, 32–43. doi: 10.1016/j.mran.2017.01.002.

- Schriewer, A., Goodwin, K. D., Sinigalliano, C. D., Cox, A. M., Wanless, D., Bartkowiak, J., Ebentier, D. L., Hanley, K. T., Ervin, J., Deering, L. A., Shanks, O. C., Peed, L. A., Meijer, W. G., Griffith, J. F., SantoDomingo, J., Jay, J. A., Holden, P. A., & Wuertz, S. (2013). Performance evaluation of canine-associated Bacteroidales assays in a multi-laboratory comparison study. Water Research, 47(18), 6909–6920. https://doi.org/10.1016/j.watres.2013.03.062

- Shanks, O. C., White, K., Kelty, C. A., Sivaganesan, M., Blannon, J., Meckes, M., et al. (2010). Performance of PCR-based assays targeting Bacteroidales genetic markers of human fecal pollution in sewage and fecal samples. *Environ. Sci. Technol.* 44, 6281–6288. doi:10.1021/es100311n.

- Sinigalliano, C. D., Ervin, J. S., van de Werfhorst, L. C., Badgley, B. D., Ballesté, E., Bartkowiak, J., Boehm, A. B., Byappanahalli, M., Goodwin, K. D., Gourmelon, M., Griffith, J., Holden, P. A., Jay, J., Layton, B., Lee, C., Lee, J., Meijer, W. G., Noble, R., Raith, M., . . . Santo Domingo, J. W. (2013). Multi-laboratory evaluations of the performance of Catellicoccus marimammalium PCR assays developed to target gull fecal sources. Water Research, 47(18), 6883–6896. https://doi.org/10.1016/j.watres.2013.02.059

- Soller, J. A., Eftim, S. E., Warren, I., and Nappier, S. P. (2017). Evaluation of microbiological risks associated with direct potable reuse. *Microb. Risk Anal.* 5, 3–14. doi:10.1016/j.mran.2016.08.003.

- Stampi, S., Varoli, O., Zanetti, F., and de Luca, G. (1993). Arcobacter cryaerophilus and thermophilic campylobacters in a sewage treatment plant in Italy: Two secondary treatments compared. 759 *Epidemiol. Infect.* 110, 633–639. doi:10.1017/S0950268800051050

  U.S. EPA (2015). Method 1611.1: Enterococci in Water by TaqMan® Quantitative Polymerase Chain Reaction (qPCR). Washington, D.C. Available at: https://www.epa.gov/sites/default/files/2015-08/documents/method_1611-1-enterococcus_2015.pdf

- U.S. EPA (2019a). Method 1696: Characterization of Human Fecal Pollution in Water by HF183/BacR287 TaqMan Quantitative Polymerase Chain Reaction (qPCR) Assay. Washington,

D.C. Available at: https://www.epa.gov/sites/default/files/2019-03/documents/method_1696_draft_2019.pdf

- U.S. EPA (2019b). Method 1697: Characterization of Human Fecal Pollution in Water by HumM2 TaqMan Quantitative Polymerase Chain Reaction (qPCR) Assay. Washington, D.C. Available at: https://www.epa.gov/sites/default/files/2019-03/documents/method_1697_draft_2019.pdf

- Yang, J., Schneider, O. D., Jjemba, P. K., and Lechevallier, M. W. (2015). Microbial Risk Modeling for Main Breaks. *Journal AWWA* 107, E97–E108. doi: 10.5942/jawwa.2015.107.0010.

2 Supplementary Data

2.1 Supplementary Figures

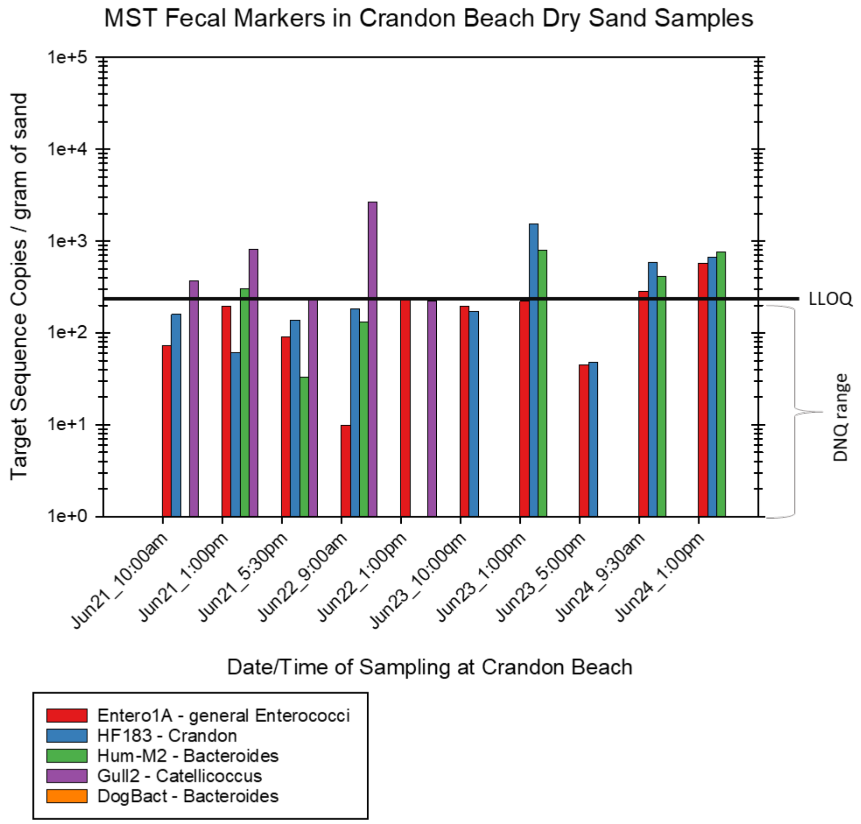

Figure S1. Abundance by date and time of MST host-source-specific fecal bacterial gene markers in Dry Sand at Crandon Park Beach as measured by target-specific qPCR. The black line labeled "LLOQ" indicates the environmental Lower Limit of Quantitation of 250 target copies/g for the listed MST gene markers. The region between the Limit of Detection (LOD) and the LLOQ is classified as DNQ ("Detected but Not Quantified") and should be considered as uncertain.

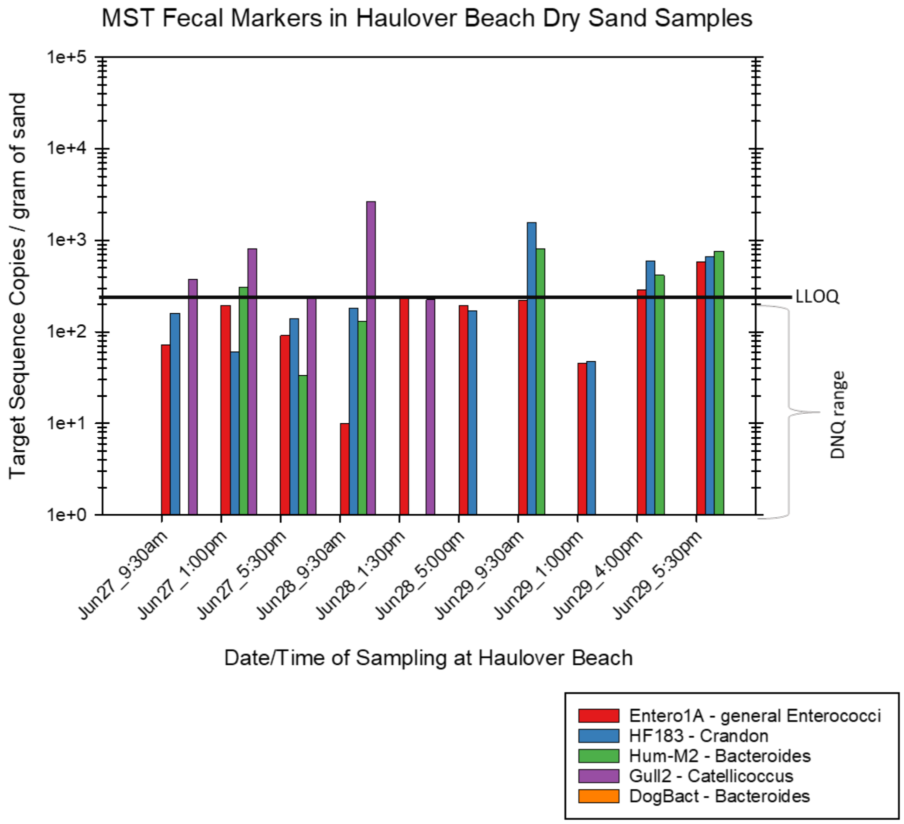

Figure S2. Abundance by date and time of MST host-source-specific fecal bacterial gene markers in Dry Sand at Haulover Beach as measured by target-specific qPCR. The black line labeled "LLOQ" indicates the environmental Lower Limit of Quantitation of 250 target copies/g for the listed MST gene markers. The region between the Limit of Detection (LOD) and the LLOQ is classified as DNQ ("Detected but Not Quantified") and should be considered as uncertain.

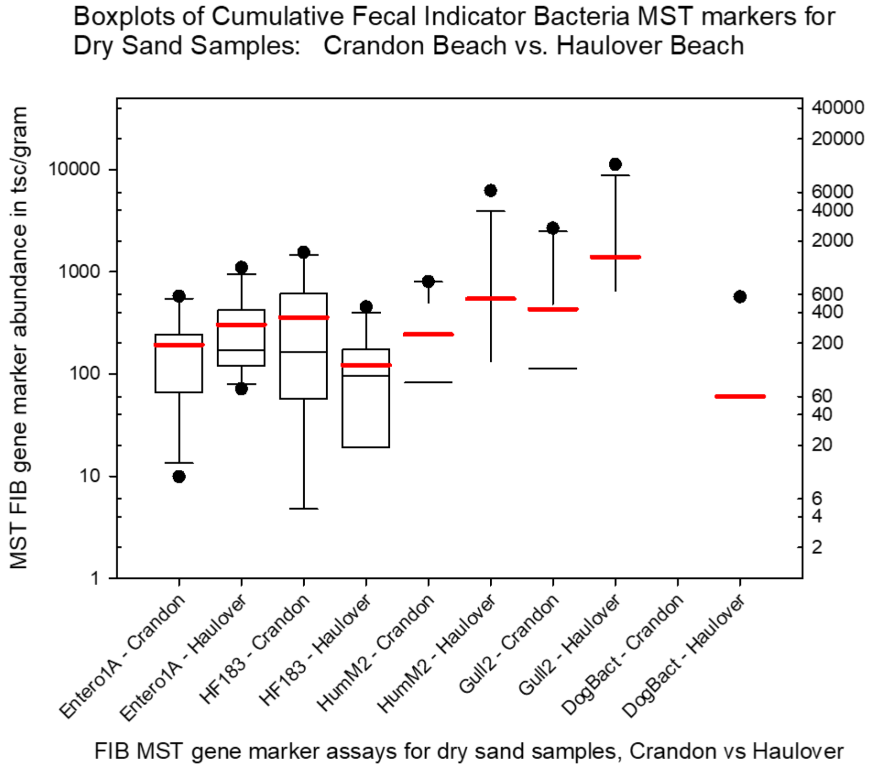

Figure S3. Boxplots of the statistical distribution of the cumulative abundance for all dry sand sample dates/times of each of the 5 MST fecal bacteria markers as measured by qPCR for Crandon Beach and Haulover Beach. The red bar associated with each plot marks the mean. Note that for some combinations of marker/beach the range between the 25th and 75th percentiles is too narrow to generate a visible box at this scale, and is just indicated by the median bar.

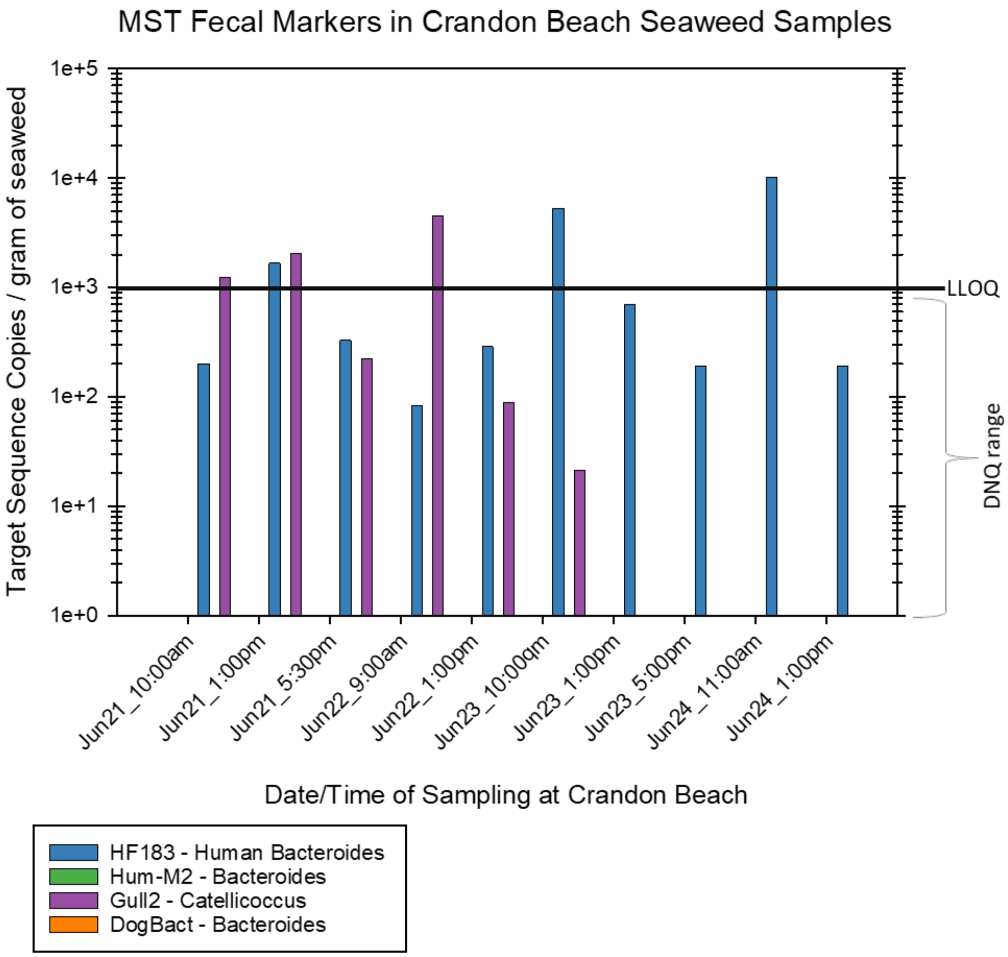

Figure S4. Abundance by date and time of MST host-source-specific fecal bacterial gene markers in seaweed wrack samples at Crandon Park Beach as measured by target-specific qPCR. The black line labeled "LLOQ" indicates the environmental Lower Limit of Quantitation of 1000 target copies/g for the listed MST gene markers. The region between the Limit of Detection (LOD) and the LLOQ is classified as DNQ ("Detected but Not Quantified") and should be considered as uncertain.

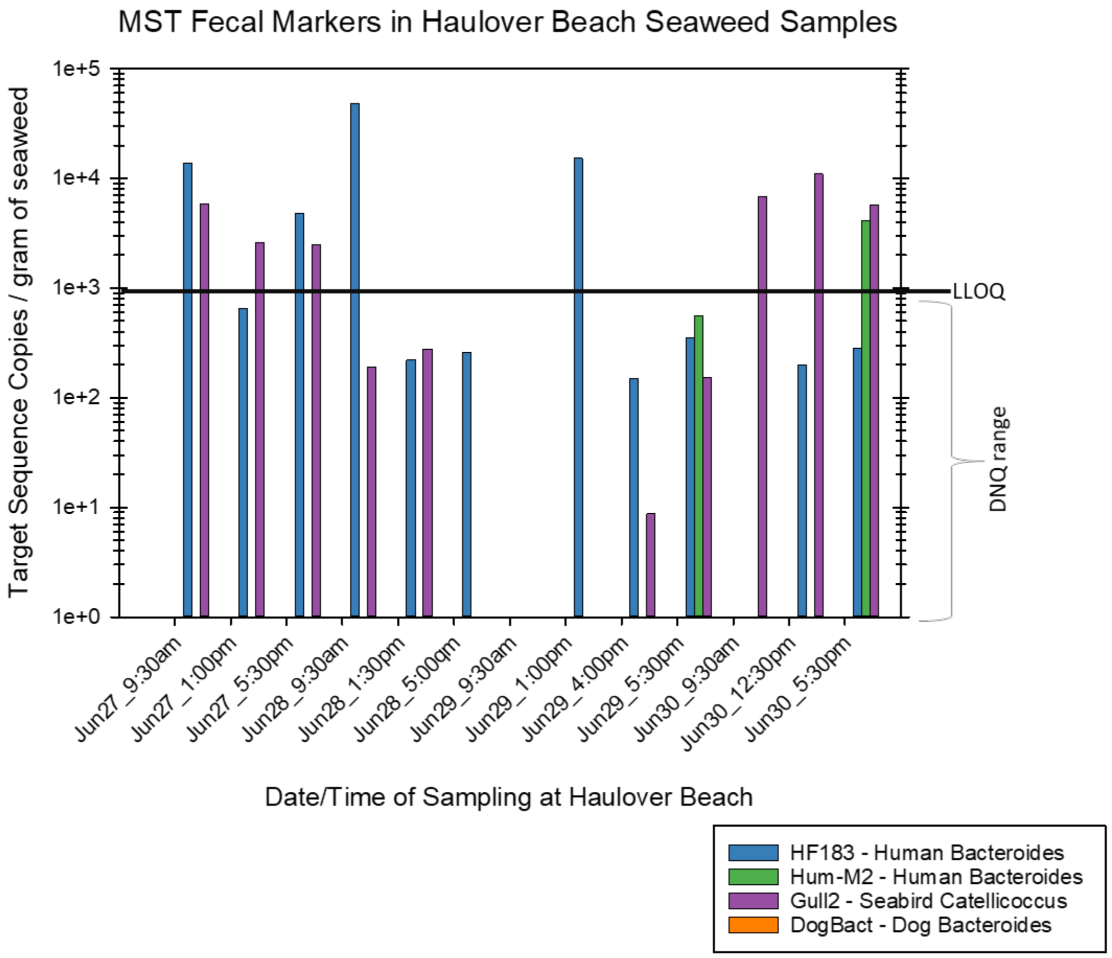

Figure S5. Abundance by date and time of MST host-source-specific fecal bacterial gene markers in seaweed wrack samples at Crandon Park Beach as measured by target-specific qPCR. The black line labeled "LLOQ" indicates the environmental Lower Limit of Quantitation of 1000 target copies/g for the listed MST gene markers. The region between the Limit of Detection (LOD) and the LLOQ is classified as DNQ ("Detected but Not Quantified") and should be considered as uncertain.

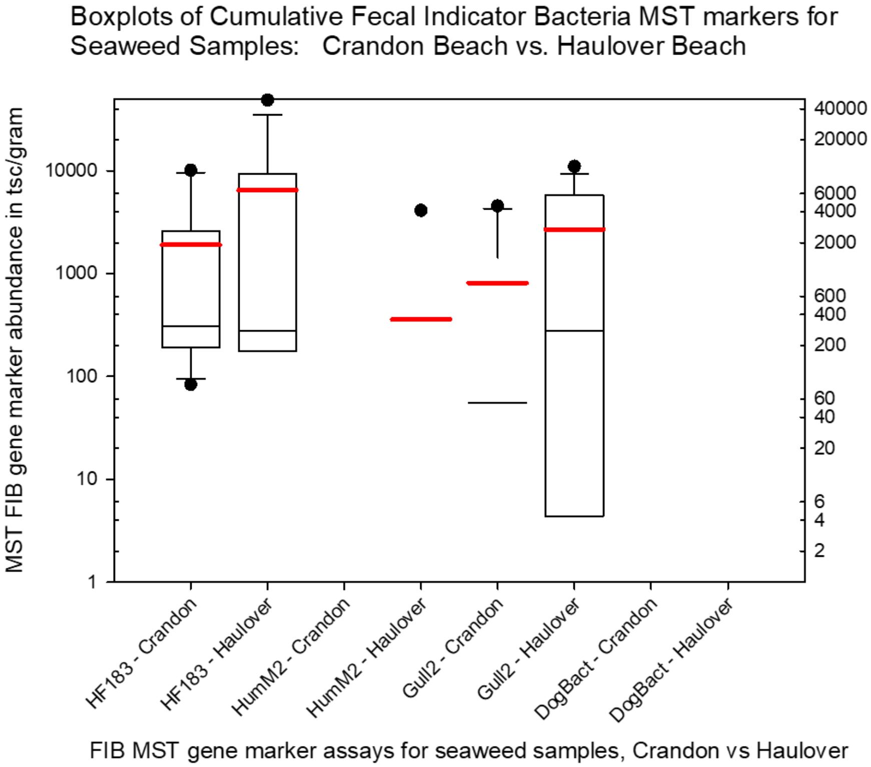

Figure S6. Boxplots of the statistical distribution of the cumulative abundance for all seaweed wrack sample dates/times of each of the 5 MST fecal bacteria markers as measured by qPCR for Crandon Beach vs Haulover Beach. The red bar associated with each plot marks the mean. Note that for some combinations of marker/beach the range between the 25th and 75th percentiles is too narrow to generate a visible box at this scale, and is just indicated by the median bar.

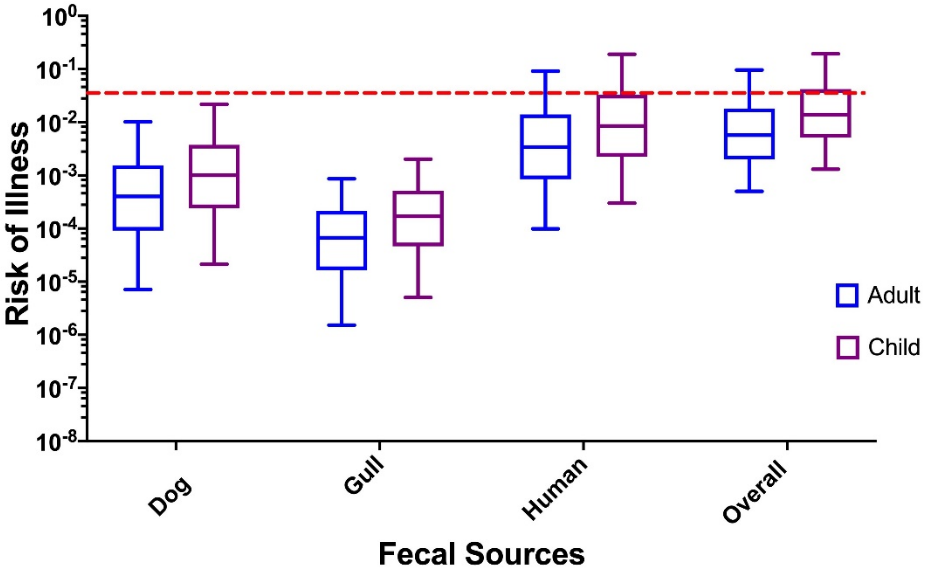

Figure S7. The risk of a GI illness per each fecal source at Haulover Beach (MST marker concentrations estimated using the DL approach). The dashed red line indicates the U.S. EPA risk threshold of 0.032.

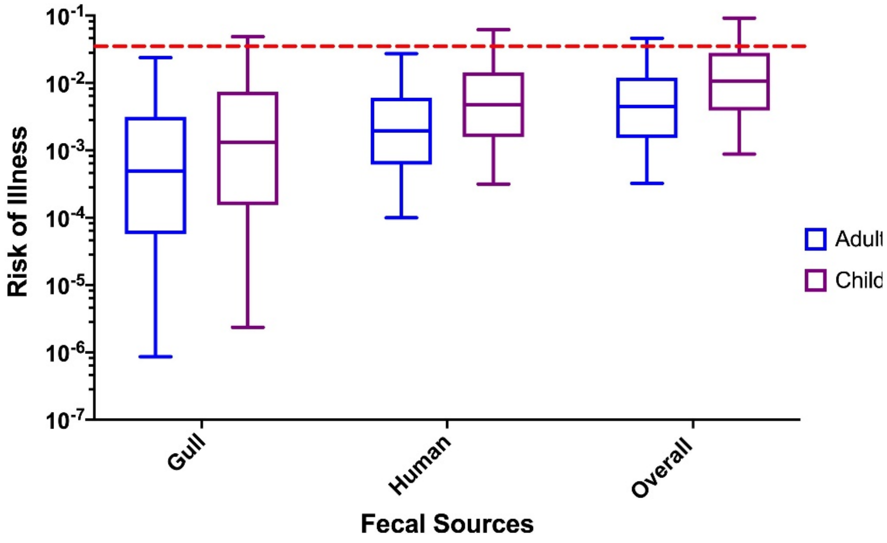

Figure S8. The risk of a GI illness per each fecal source at Crandon Beach (without the dog fecal source present). MST marker concentrations were estimated using the DL approach. The dashed red line indicates the U.S. EPA risk threshold of 0.032.

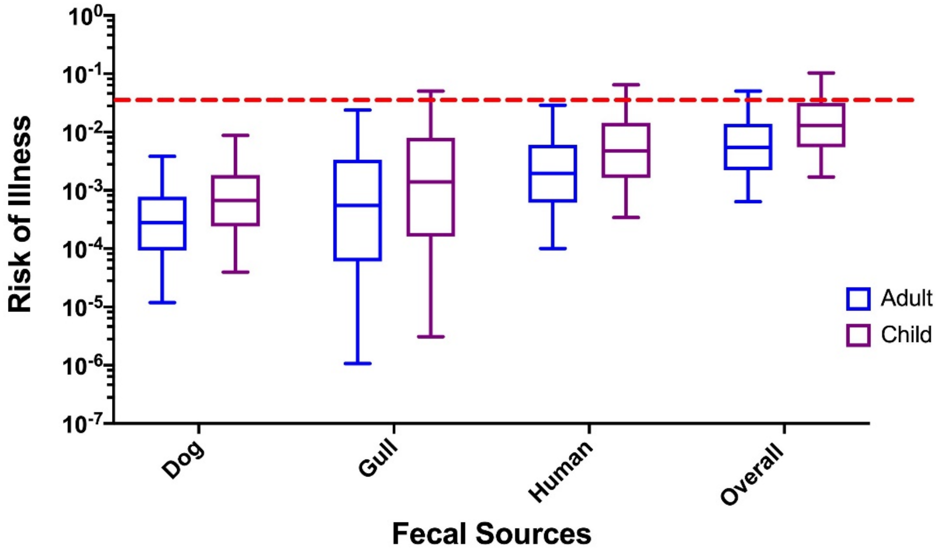

Figure S9. The risk of a GI illness per each fecal source at Crandon Beach (HF183 and Gull2 marker concentrations were estimated using the DL approach). DogBact marker concentration was assumed to be 25 copies/100 mL. The dashed red line indicates the U.S. EPA risk threshold of 0.032.

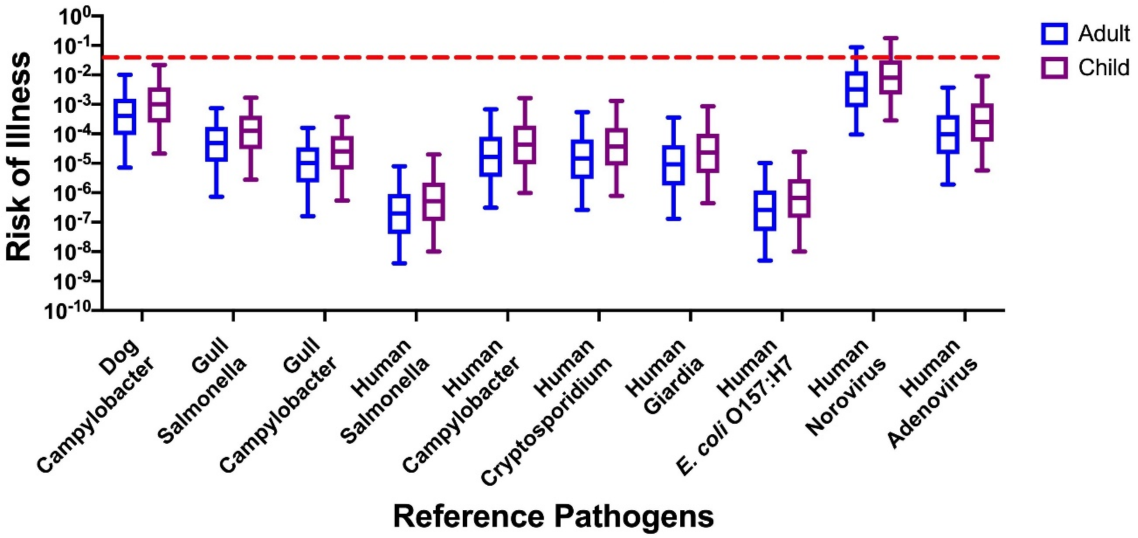

Figure S10. The risk of illness associated with each reference pathogen at Haulover Beach (MST marker concentrations estimated using the DL method). The dashed red line indicates the U.S. EPA risk threshold of 0.032.

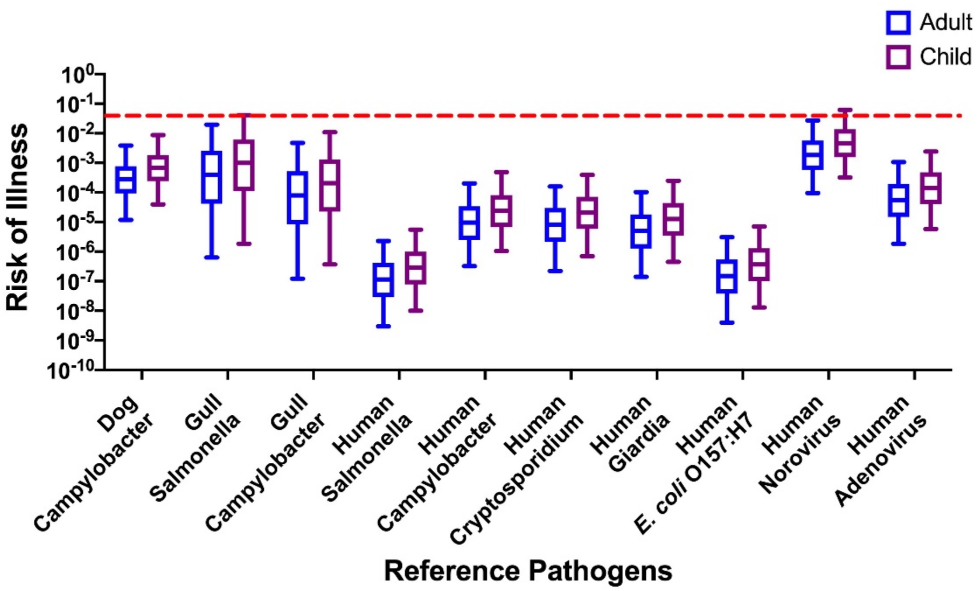

Figure S11. The risk of illness associated with each reference pathogen at Crandon Beach. The DogBact MST marker concentration was assumed to be 25 copies/100 mL (HF183 and Gull2 marker concentrations were estimated using the DL method). The dashed red line indicates the U.S. EPA risk threshold of 0.032.

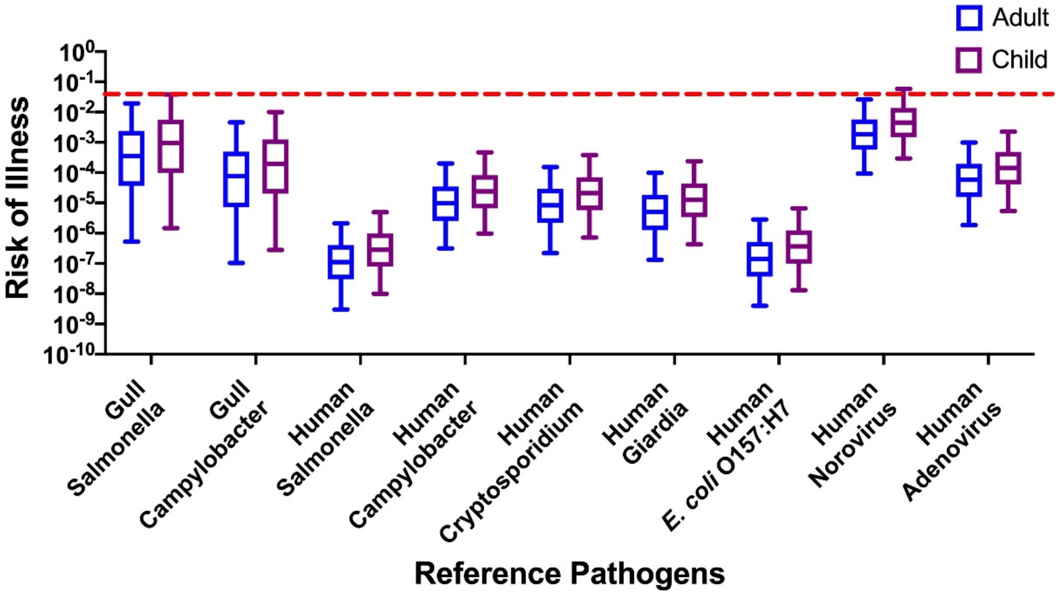

Figure S12. The risk of illness associated with each reference pathogen at Crandon Beach assuming no dog fecal source was present. HF183 and Gull2 marker concentrations were estimated using the DL approach. The dashed red line indicates the U.S. EPA risk threshold of 0.032.

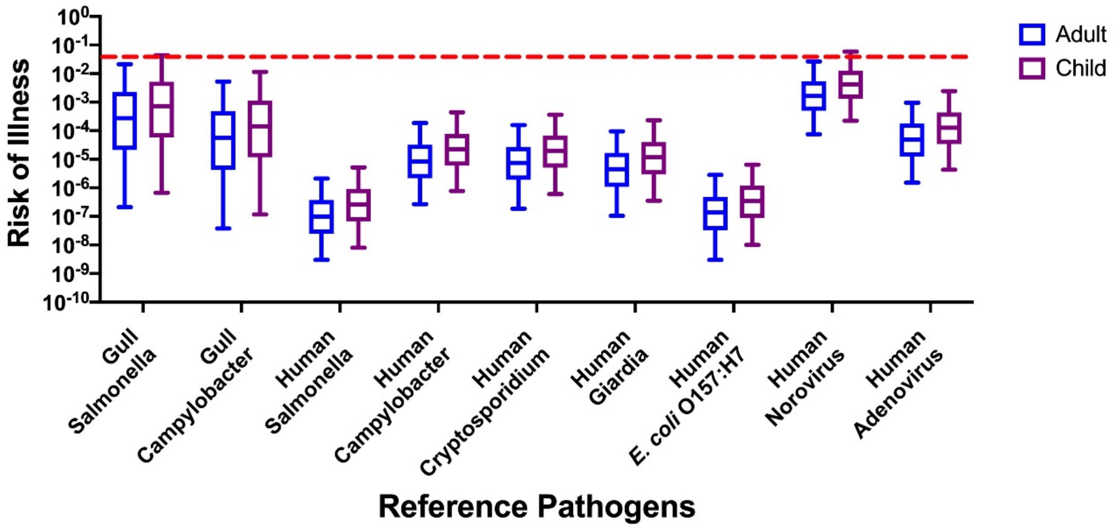

Figure S13. The risk of illness associated with each reference pathogen at Crandon Beach, assuming no dog fecal source was present. HF183 and Gull2 marker concentrations were estimated using the INT method. The dashed red line indicates the U.S. EPA risk threshold of 0.032**.**

2.2 Supplementary Tables

Table S1. Primers, probes, and gBlock positive control sequences for qPCR assays

Host/Assay Name	Oligonucleotide Primer/Probe sequences & gBlock Positive Control sequences* (sequence 5'→ 3')
**HUMAN:**<br>HF183 Taqman<br><br>(EPA version HF183/BacR287)	**Target organism:** Bacteroides 16S rRNA gene. **REF:** Griffith et al. (2013); U.S. EPA Method 1696 (2019).<br>Forward Primer: HF183. ATCATGAGTTCACATGTCCG<br>Reverse Primer: BacR287. CTTCCTCTCAGAACCCCTATCC<br>Probe: BacP234MGB. [6FAM]-CTAATGGAACGCATCCC-[NFQ-MGB]<br>HF183 gBlock positive control:<br>ATCATGAGTTCACATGTCCGCATGATTAAAGGTATTTTCCGGTAGACGATGGGGATGCGTTCCATTAGCTCGAGATAGTAGGCGGGGTAACG<br>GCCCACCTAGTCAACGATGGATAGGGGTTCTGAGAGGAAGG
**HUMAN:**<br>HumM2	**Target organism:** Bacteroides Cell surface protein genes. **REF:** U.S..EPA Method 1697 (2019).<br>Forward Primer: HumM2F. CGTCAGGTTTGTTTCGGTATTG<br>Reverse Primer: HumM2R. TCATCACGTAACTTATTTATATGCATTAGC<br>Probe: HumM2P. [6-FAM]-TATCGAAAATCTCACGGATTAACTCTTG TGTACGC-TAMRA<br>HumM2 gBlock positive control:<br>CGTCAGGTTTGTTTCGGTATTGAGTATCGAAAATCTCACGGATTAACTCTTGTGTACGCTCTCGAGGACCAGCTAATGCATATAAATAAGTT<br>ACGTG
**SEABIRD:**<br>Gull2	**Target organism:** *Catellicoccus marimammalium* 16S rRNA gene. **REF:** Griffith et al. (2013); Sinigalliano et al. (2013).<br>Forward Primer: Gull2F TGCATCGACCTAAAGTTTTGAG<br>Reverse Primer: Gull2R GTCAAAGAGCGAGCAGTTACTA<br>Probe: Gull2P. [6FAM]-CTGAGAGGGTGATCGGCCACATTGGGACT-[BHQ1]<br>Gull2 gBlock positive control:<br>TAATACATGCAAGTCGAACGCAAAACTTTTAACTGATGCTTGCATCGACCTAAAGTTTTGAGTGGCGGACGGGTGAGTAACACGTGGGTAA<br>CCTGCCCATCAGAGGGGGACAACACTTGGAAACAGGTGCTAATACCGCATAATACAGAGAACCGCATGGTTCTTTGTTGAAAGGCGCTTCT<br>GGTGTCGCTGATGGATGGACCCGCGGTGCATTAGCTAGACGGTGAGGTAACGGCTCACCGTGGCAATGATGCATAGCCGACCTGAGAGGGT<br>GATCGGCCACATTGGGACTGAGACACGGCCCAAACTCCTACGGGAGGCAGCAGTAGGGAATCTTCGGCAATGGACGAAAGTCTGACCGAG<br>CAACGCCGCGTGAGTGAAGAAGGTTTTCGGATCGTAAAACTCTGTTGTTAGAGAAGAACAGGAGCGATAGTAACTGCTCGCTCTTTGACGG<br>TATCTAACCAGAAAGCCACGGCTAACTACGTGCCAGCAGCCGCGGTAATACGTAGGTGGCAAGCGTTGTCCGGATTTATTGGGCGTAAAGC<br>GAGCGCAGGCGGTC
**DOG:**<br>DogBact	**Target organism:** Bacteroides 16S rRNA gene. **REF:** Griffith et al. (2013); Schriewer et al. (2013).<br>Forward Primer: DF475F. CGCTTGTATGTACCGGTACG<br>Reverse Primer: Bac708R. CAATCGGAGTTCTTCGTG<br>Probe: DogBactP. [6FAM]-ATTCGTGGTGTAGCGGTGAAATGCTTAG-[BHQ1]<br>DogBact gBlock positive control:<br>CTTTTGTCCGGGAATAAAACCGCCTACGTGTAGGCGCTTGTATGTACCGGTACGAATAAGCATCGGCTAACTCCGTGCCAGCAGCCGCGGTA<br>ATACGGAGGATGCGAGCGTTATCCGGATTTATTGGGTTTAAAGGGAGCGCAGACGGGTTTTTAAGTCAGCTGTGAAAGTTTGGGGCTCAAC<br>CTTAAAATTGCAGTTGATACTGGAGACCTTGAGTGCAGTTGAGGCAGGCGGAATTCGTGGTGTAGCGGTGAAATGCTTAGATATCACGAAG<br>AACTCCGATTGCGAAGGCAGCTTGCTAAAGTGTAACTGACGTTCATGCTCGAAAGTGTGGGTATCAAACAGGATTAGATACCCTGG

ENTEROCOCCI: Entero1A	Target organism: *Enterococcus*. **REF:** U.S. EPA Method 1611.1 (2015)	
	Forward Primer:	GAGAAATTCCAAACGAACTTG
	Reverse Primer:	CAGTGCTCTACCTCCATCATT
	Probe:	[6-FAM]-TGGTTCTCTCCGAAATAGCTTTAGGGCTA-TAMRA

* All primer, probe, and gBlock oligonucleotide sequences were synthesized by Integrated DNA Technologies (IDTDNA.com), except for the minor-groove-binding probes using NFG-MGB quenchers, which were synthesized by Applied Biosystems-Life Technologies (www.FisherSci.com

Table S2. Mean quality control parameters of qPCR for MST assays performed

qPCR Assay	Mean R^2	Mean Y intercept	Mean Slope	Mean LLOQ (Cq) (upper 95th prediction interval of 1log10 std)	Mean LLOQ (copies/rxn) (upper 95th prediction interval of 1log10 std)	Standardized mean LLOQ (copies/rxn) *(used for calculating eLLOQ)*	Mean E (%)	NTC (Cq)	*Calculated eLLOQ for water (copies/100mL)	*Calculated eLLOQ for sand (copies/gram)	*Calculated eLLOQ for seaweed wrack (copies/gram)
**Enterococci Entero1A**	0.991	37.918	-3.436	34.620	9.2	10	95.46	ND	50	250	1000
**Human HF183**	0.995	39.484	-3.445	36.162	9,2	10	95.12	ND	50	250	1000
**Human HumM2**	0.998	39.438	-3.397	36.611	6.8	10	96.94	ND	50	250	1000
**Seabird Gull2**	0.996	37.853	-3.403	34.674	8.6	10	96.73	ND	50	250	1000
**Dog DogBact**	0.989	39.704	-3.272	36.429	10.0	10	102.14	ND	50	250	1000

* Reaction LLOQs are measured from the upper 95% prediction interval of the 10 copy/reaction quantitation standard from the combined consensus standard curve for each assay under these particular reaction conditions. The calculated Environmental Lower Limit of Quantitation (eLLOQ) is based upon using a standardized mean LLOQ set to 10 copies/reaction for all assays, and based upon the volume of water filtered or mass of sand or seaweed extracted, the elution volume, any dilutions, and volume of target DNA added to qPCR reactions.

Table S3. Parameters and their distributions used to estimate the reference pathogen dose

Parameter	Units	Concentration	Source
**HF183 in the environment**	copies/100mL	Interval Censored: Haulover (4.38822, 2.02544)[a]; Crandon (3.78605, 1.54293)[a]<br>Substitution: Haulover (4.50538, 1.91507[a]; Crandon (3.88925, 1.46944)[a]	Environmental data
**Gull2 in the environment**	copies/100mL	Interval Censored: Haulover (3.26340, 1.39613)[a]; Crandon (679.841, 0.388813)[b]<br>Substitution: Haulover (67.31681, 0.93997)[b]; Crandon (835.67521, 0.44577)[b]	Environmental data
**DogBact in the environment**	copies/100mL	Interval Censored: Haulover (21.1045, 0.423840)[b]; Crandon 0 or 25[c]<br>Substitution Haulover (63.18327, 0.90705)[b]; Crandon 0 or 25[c]	Environmental data
**HF183 in human sewage**	copies/mL	(5.212, 0.566)[d]	(Shanks et al., 2010)
**Gull2 in gull waste**	copies/g	(8.7,8.3)[e]	(Brown et al., 2017a)[i]
**DogBact marker in dog waste**	copies/g	(5,9)[f]	(Ervin et al., 2014)
***Campylobacter* in Dog Feces**	organisms/g	(3, 8)[f]	(Chaban et al., 2010)
***Campylobacter* in Gull Feces**	CFU/g	(3.3, 6)[f]	(Lévesque et al., 2000)
***Salmonella* in Gull Feces**	CFU/g	(2.3, 9.0)[f]	(Lévesque et al., 2000)
***Salmonella* in Sewage**	CFU/L	(0.5,5)[f]	(Koivunen et al., 2003; Lemarchand and Lebaron, 2003)
***Campylobacter* in Sewage**	MPN/L	(2.9,4.6)[f]	(Stampi et al., 1993)
***E. coli* O157:H7 in sewage**	CFU/L	(-1,3.3)[f,j]	(Garcia-Aljaro et al., 2005)
***Cryptosporidium* in sewage**	oocysts/L	(-0.52, 3.7)[f]	(Harwood et al., 2005; Crockett, 2007; Yang et al., 2015; Nasser, 2016; Schoen et al., 2017)
***Giardia* in sewage**	cysts/L	(0.51, 4.2)[f]	(Harwood et al., 2005; Kitajima et al., 2014)
**Norovirus in sewage**	copy/L	(4.7, 1.5)[d]	(Eftim et al., 2017)
**Adenovirus in sewage**	IU/L	(1.75, 3.84)[f]	(Hurst et al., 1988; Hewitt et al., 2011; Soller et al., 2017)
**Volume water ingested**	adult (mL)	(32.3, 70.5)[g, k]	(DeFlorio-Barker et al., 2018)

Parameter	Units	Concentration	Source
	children (mL)	(67.7, 160)[g, l]	(DeFlorio-Barker et al., 2018)
**Fraction of Pathogenic Species**	Gull	0.01-0.4[h]	(Garcia-Aljaro et al., 2005)
	Sewage	1	Assumed
	Dog	0.02- 0.1[h]	(Gras et al., 2013)

[a] Lognormal distribution (log mean, log standard deviation); [b] Weibull distribution (scale, shape); [c] Point estimate; [d] Log_{10}-normal distribution (mean, standard deviation);[e]Log_{10}-weibull distribution (scale, shape);[f] Log_{10}-uniform distribution (minimum, maximum);[g] normal distribution (mean, 90%); [h] uniform distribution (minimum, maximum);[i] The *C. marimammalium* concentration was estimated using the LeeGull marker, but the Gull2 marker which is used in this study, identifies the same target region of *C. marimammalium*.; [j] The lower range was not detected and -1 is used as a lower bound for *E. coli* O157:H7.; [k] Ingestion value for adults age 35 and over recreating in marine water.; [l] Ingestion values for children age 6-12 recreating in marine water

Table S4. Median risk of GI illness for each reference pathogen at both Haulover and Crandon Park Beaches

		**Median Risk of Illness (INT method)**		**Median Risk of Illness (DL method)**	
**Beach**	**Reference Pathogen**	**Adult**	**Child**	**Adult**	**Child**
Haulover	Adenovirus	9.22×10^{-5}	2.24×10^{-4}	9.59×10^{-5}	2.52×10^{-4}
	Norovirus	2.97×10^{-3}	7.34×10^{-3}	3.22×10^{-3}	7.97×10^{-3}
	Campylobacter (Human)	1.49×10^{-5}	3.65×10^{-5}	1.64×10^{-5}	4.31×10^{-5}
	Cryptosporidium	1.35×10^{-5}	3.35×10^{-5}	1.46×10^{-5}	3.69×10^{-5}
	Giardia	8.26×10^{-6}	2.00×10^{-5}	9.21×10^{-6}	2.297×10^{-5}
	E. coli O157:H7	3.00×10^{-9}	5.75×10^{-7}	2.60×10^{-7}	6.60×10^{-7}
	Salmonella (Human)	2.00×10^{-9}	4.47×10^{-7}	4.00×10^{-8}	5.20×10^{-7}
	Campylobacter (Dog)	7.96×10^{-5}	2.10×10^{-4}	4.03×10^{-4}	1.01×10^{-3}
	Campylobacter (Gull)	6.41×10^{-6}	1.62×10^{-5}	1.01×10^{-5}	2.53×10^{-5}
	Salmonella (Gull)	6.51×10^{-7}	7.96×10^{-5}	4.84×10^{-5}	1.25×10^{-4}
Crandon (excluding Dog)	Adenovirus	4.92×10^{-5}	1.27×10^{-4}	5.83×10^{-5}	1.44×10^{-4}
	Norovirus	1.64×10^{-3}	4.20×10^{-3}	1.84×10^{-3}	4.50×10^{-3}
	Campylobacter (Human)	8.40×10^{-6}	2.21×10^{-5}	9.71×10^{-6}	2.41×10^{-5}
	Cryptosporidium	7.49×10^{-6}	1.98×10^{-5}	8.38×10^{-6}	2.10×10^{-5}
	Giardia	4.49×10^{-6}	1.19×10^{-5}	5.07×10^{-6}	1.26×10^{-5}
	E. coli O157:H7	1.37×10^{-7}	3.42×10^{-7}	1.41×10^{-7}	3.65×10^{-7}
	Salmonella (Human)	9.90×10^{-8}	2.59×10^{-7}	1.10×10^{-7}	2.83×10^{-7}
	Campylobacter (Gull)	5.64×10^{-5}	1.43×10^{-4}	7.60×10^{-5}	1.97×10^{-4}
	Salmonella (Gull)	2.76×10^{-4}	7.26×10^{-4}	3.53×10^{-4}	9.52×10^{-4}
Crandon (with Dog)	Adenovirus	4.92×10^{-5}	1.25×10^{-4}	5.53×10^{-5}	1.40×10^{-4}
	Norovirus	1.61×10^{-3}	4.06×10^{-3}	1.85×10^{-3}	4.55×10^{-3}
	Campylobacter (Human)	8.24×10^{-6}	2.16×10^{-5}	9.63×10^{-6}	2.39×10^{-5}
	Cryptosporidium	7.25×10^{-6}	1.84×10^{-5}	8.22×10^{-6}	2.07×10^{-5}
	Giardia	4.44×10^{-6}	1.12×10^{-5}	5.03×10^{-6}	1.27×10^{-5}
	E. coli O157:H7	1.27×10^{-7}	3.23×10^{-7}	1.49×10^{-7}	3.69×10^{-7}
	Salmonella (Human)	1.00×10^{-7}	2.49×10^{-7}	1.14×10^{-7}	2.88×10^{-7}
	Campylobacter (Dog)	2.78×10^{-4}	6.93×10^{-4}	2.77×10^{-4}	6.73×10^{-4}
	Campylobacter (Gull)	5.55×10^{-5}	1.44×10^{-4}	7.97×10^{-5}	2.07×10^{-4}
	Salmonella (Gull)	2.74×10^{-4}	7.07×10^{-4}	3.97×10^{-4}	9.98×10^{-4}

2.3 Sensitivity Analyses for Human Health Risk Outputs

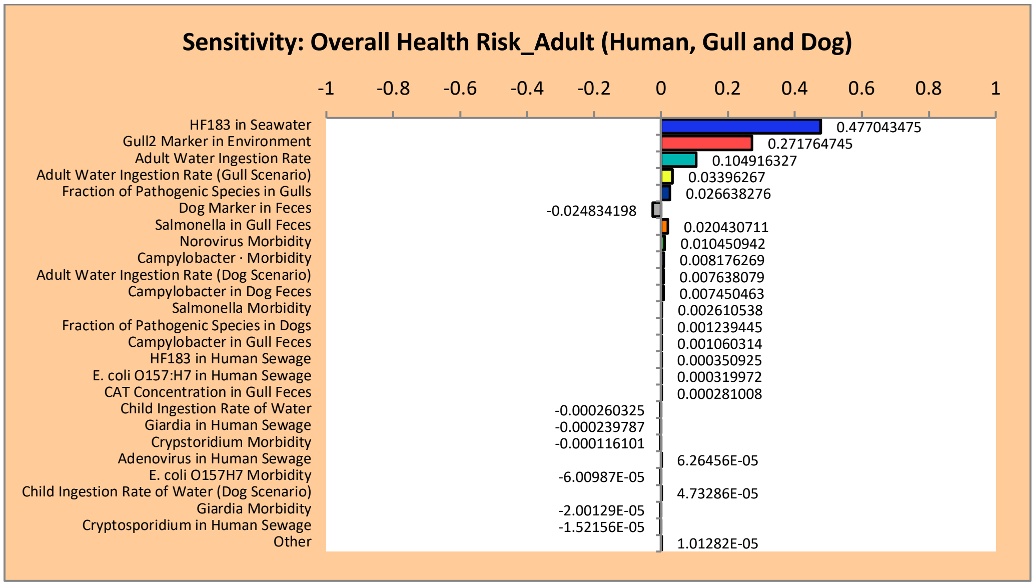

Figure S14. Sensitivity analysis of the overall adult human health risk output for Crandon Beach (MST marker concentrations estimated using the DL method) assuming the dog fecal source was present.

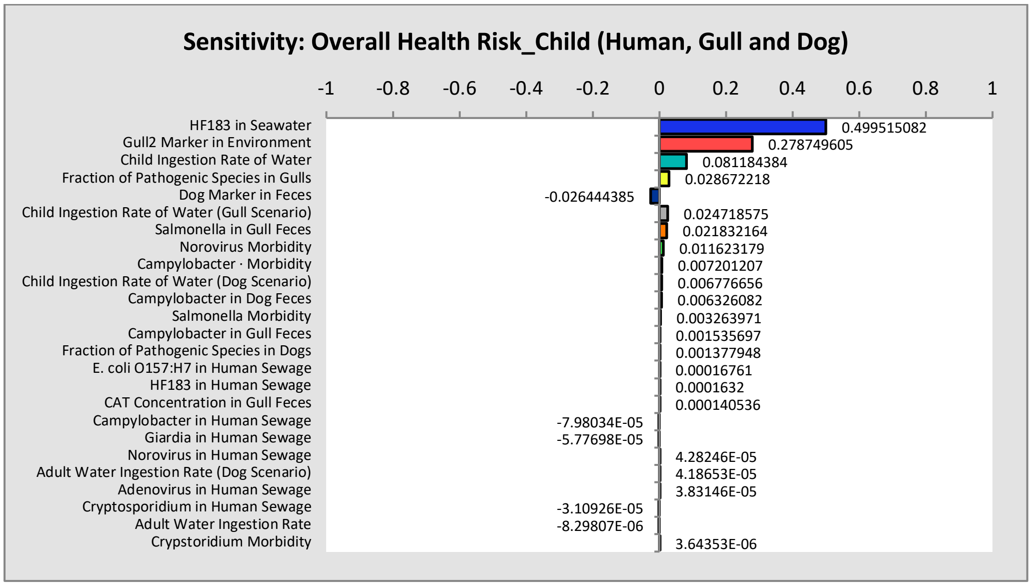

Figure S15. Sensitivity analysis of the overall child human health risk output for Crandon Beach (MST marker concentrations estimated using the DL method) assuming the dog fecal source was present.

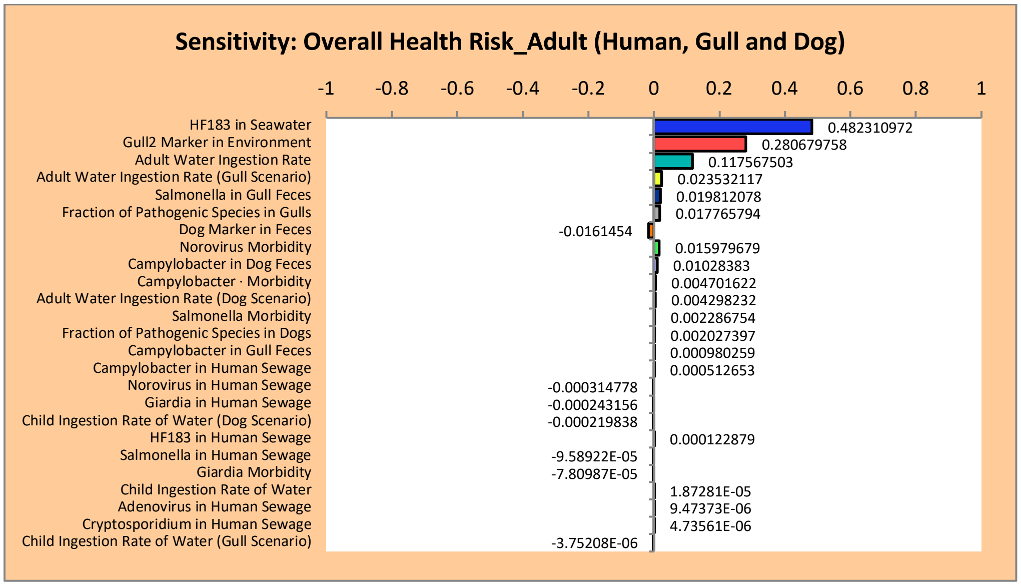

Figure S16. Sensitivity analysis of the overall adult human health risk output for Crandon Beach (MST marker concentrations estimated using the INT method) assuming the dog fecal source was present.

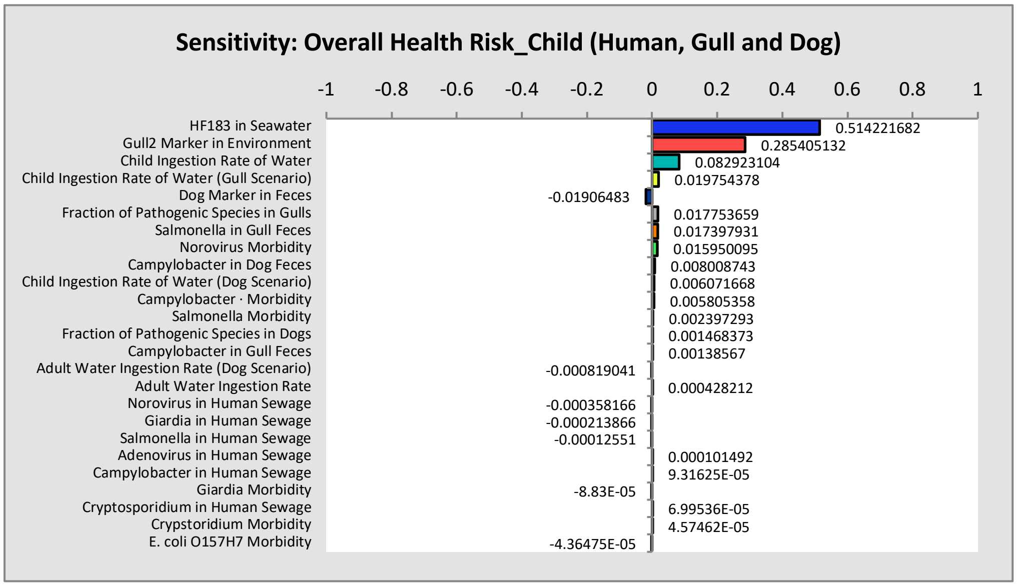

Figure S17. Sensitivity analysis of the overall child human health risk output for Crandon Beach (MST marker concentrations estimated using the INT method) assuming the dog fecal source was present.

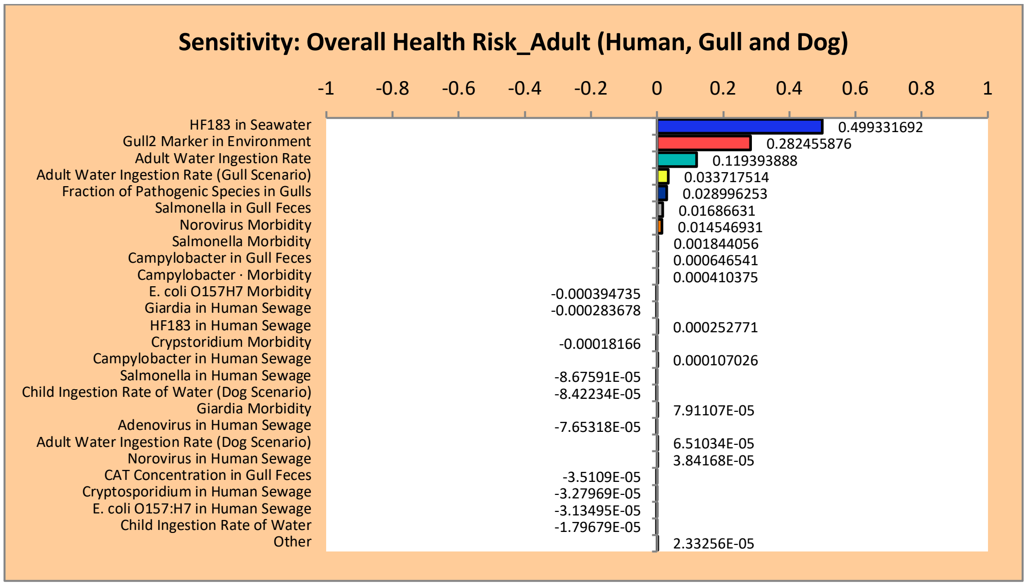

Figure S18. Sensitivity analysis of the overall adult human health risk output for Crandon Beach (MST marker concentrations estimated using the DL method) assuming no dog fecal source was present.

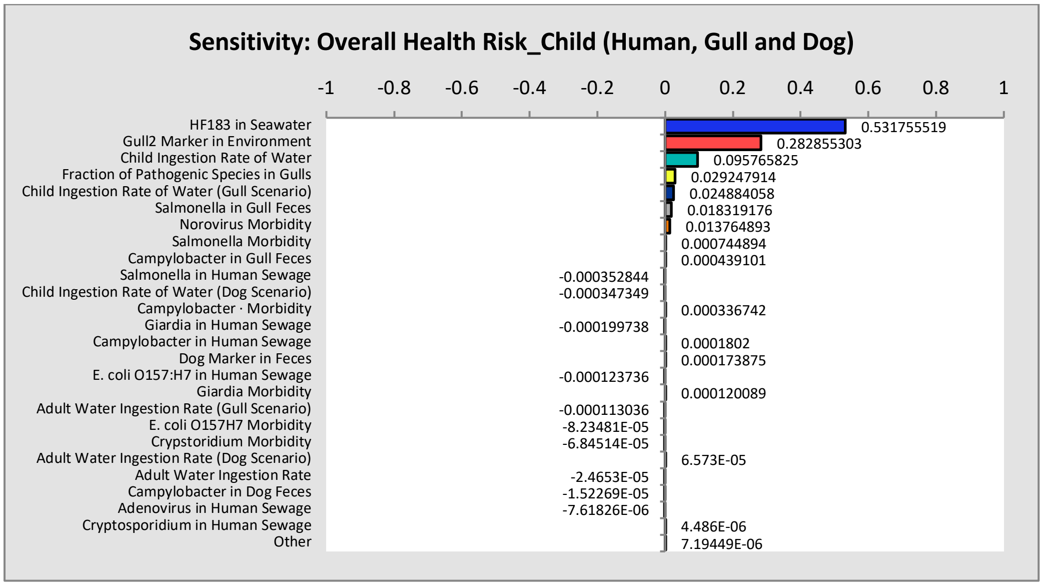

Figure S19. Sensitivity analysis of the overall child human health risk output for Crandon Beach (MST marker concentrations estimated using the DL method) assuming no dog fecal source was present.

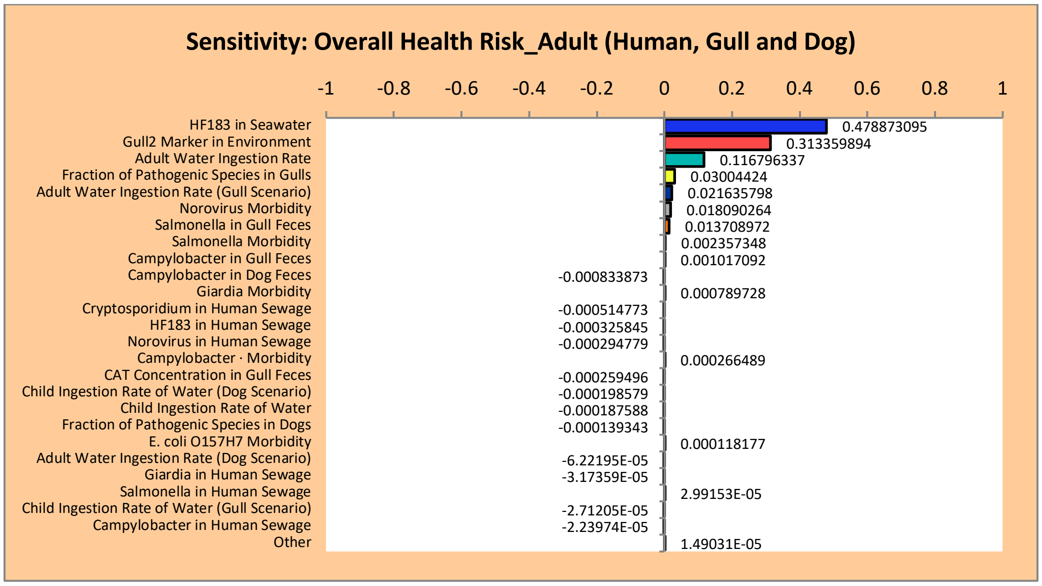

Figure S20. Sensitivity analysis of the overall adult human health risk output for Crandon Beach (MST marker concentrations estimated using the INT method) assuming no dog fecal source was present.

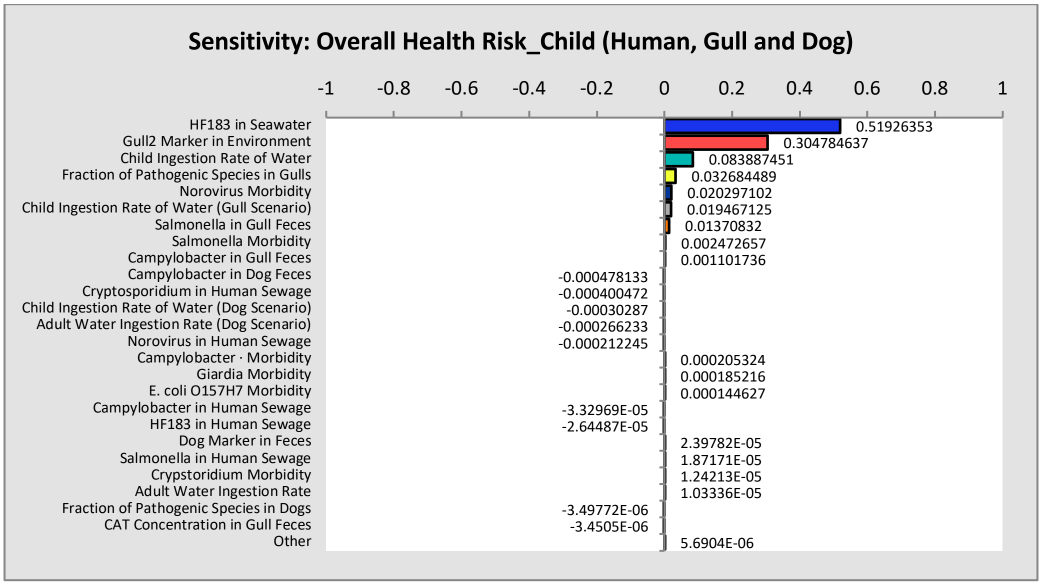

Figure S21. Sensitivity analysis of the overall child human health risk output for Crandon Beach (MST marker concentrations estimated using the INT method) assuming no dog fecal source was present.

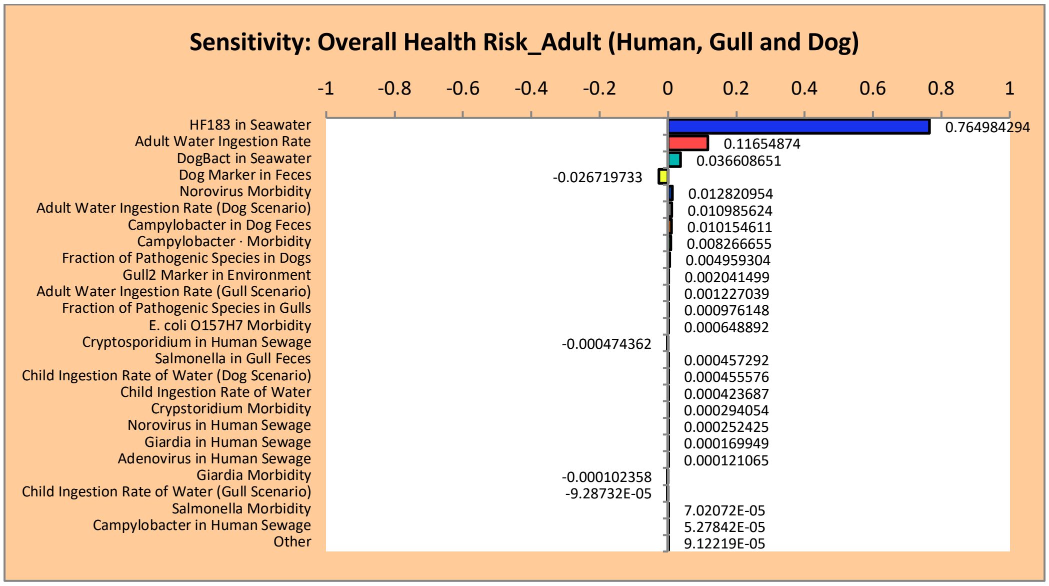

Figure S22. Sensitivity analysis of the overall adult human health risk output for Haulover Beach (MST marker concentrations estimated using the DL method).

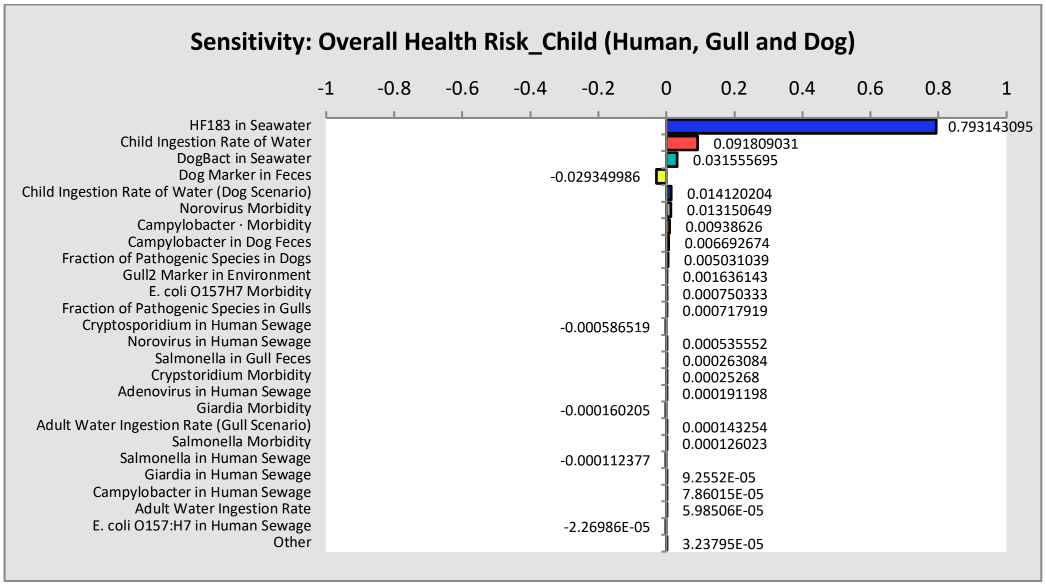

Figure S23. Sensitivity analysis of the overall child human health risk output for Haulover Beach (MST marker concentrations estimated using the DL method).

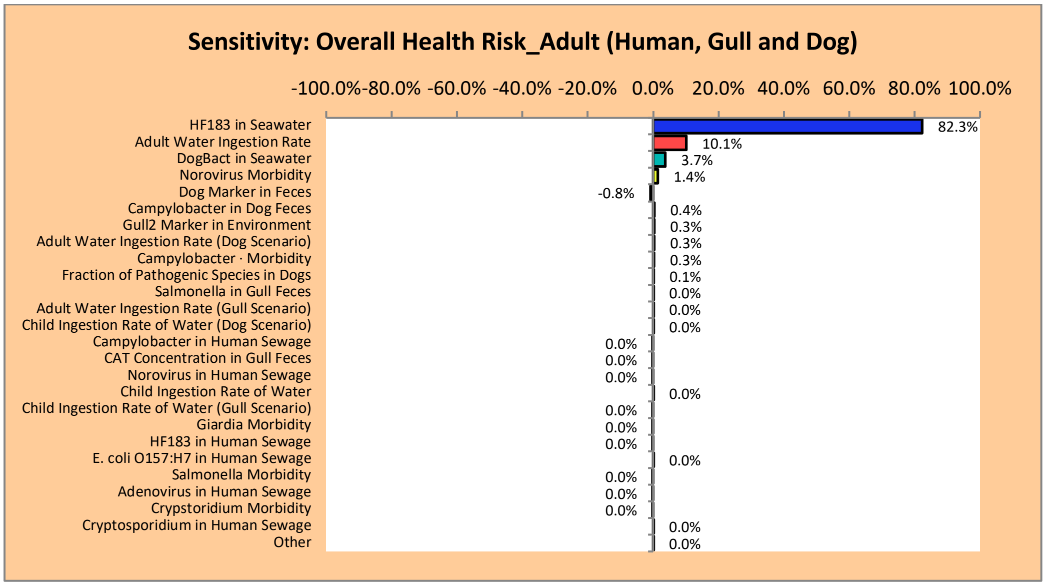

Figure S24. Sensitivity analysis of the overall adult human health risk output for Haulover Beach (MST marker concentrations estimated using the INT method).

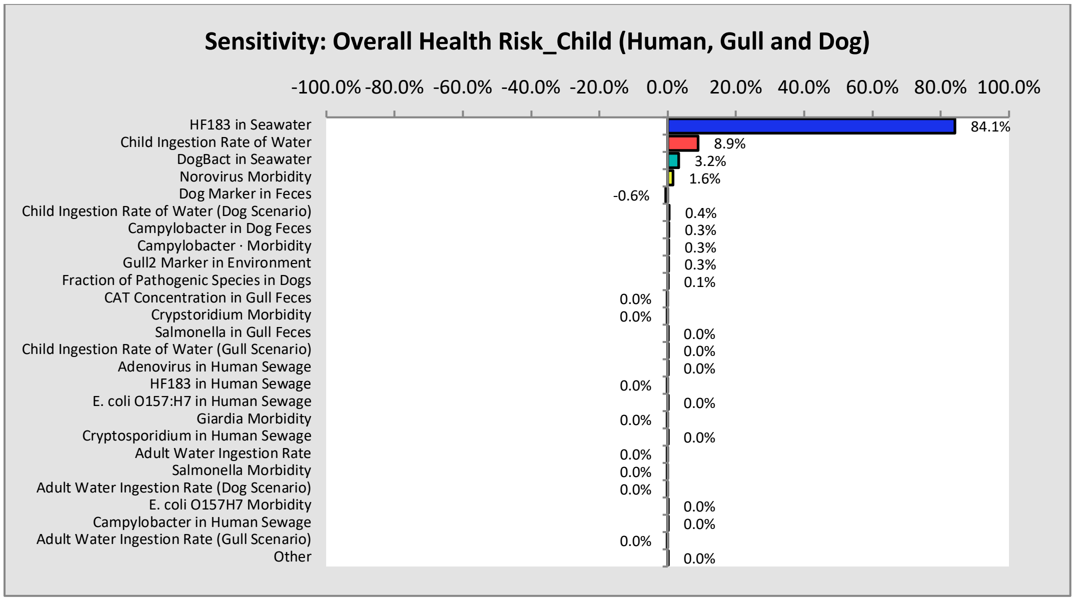

Figure S25. Sensitivity analysis of the overall child human health risk output for Haulover Beach (MST marker concentrations estimated using the INT method).
